# OTUD5 Protects Dopaminergic Neurons by Promoting the Degradation of α‐Synuclein in Parkinson's Disease Model

**DOI:** 10.1002/advs.202406700

**Published:** 2024-12-25

**Authors:** Xiaomeng Song, Tengfei Liu, Lu Yu, Qiuran Ji, Xin Guo, Runzhe Zong, Yiquan Li, Gan Huang, Qidi Xue, Qingyi Fu, Bingyu Liu, Yi Zheng, Lin Chen, Chengjiang Gao, Huiqing Liu

**Affiliations:** ^1^ Department of Pharmacology School of Basic Medical Sciences Shandong University Jinan Shandong 250012 P. R. China; ^2^ Key Laboratory of Infection and Immunity of Shandong Province School of Basic Medical Sciences Shandong University Jinan Shandong 250012 P. R. China; ^3^ Department of Immunology School of Basic Medical Sciences Shandong University Jinan Shandong 250012 P. R. China; ^4^ Department of Rehabilitation Medicine The Second Hospital Shandong University Jinan Shandong 250012 P. R. China

**Keywords:** deubiquitinase, endolysosomal pathway, nedd4, otud5, parkinson's disease, α‐synuclein

## Abstract

Defective clearance and accumulation of α‐synuclein (α‐Syn) is the key pathogenic factor in Parkinson's disease (PD). Recent studies emphasize the importance of E3 ligases in regulating the degradation of α‐Syn. However, the molecular mechanisms by which deubiquitinases regulate α‐Syn degradation are scarcely studied. In this study, it is found that the protein levels of α‐Syn are negatively regulated by ovarian tumor protease deubiquitinase 5 (OTUD5) which protects dopaminergic (DA) neurons in the PD model. Mechanistically, OTUD5 promotes K63‐linked polyubiquitination of α‐Syn independent of its deubiquitinating enzyme activity and mediates its endolysosomal degradation by recruiting the E3 ligase neural precursor cell expressed developmentally downregulated 4 (NEDD4). Furthermore, OTUD5 conditional knockout in DA neurons results in more severe α‐Syn related pathology and dyskinesia after injection of α‐Syn preformed fibrils (PFF). Overall, the data unveil a novel mechanism to regulate the degradation of α‐Syn and provide a new therapeutic strategy to alleviate DA neurodegeneration.

## Graphical abstract

Ovarian tumor protease deubiquitinase 5 (OTUD5) downregulates α‐synuclein (α‐Syn) protein levels and protects dopaminergic (DA) neurons in Parkinson's disease (PD) model. Mechanistically, OTUD5 promotes K63‐linked polyubiquitination and endolysosomal degradation of α‐Syn by recruiting the E3 ligase neural precursor cell expressed developmentally downregulated 4 (NEDD4). Overall, a novel mechanism to regulate the degradation of α‐Syn in the PD pathological process is revealed.

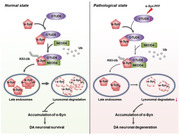

## 1 Introduction

Parkinson's disease (PD) is one of the most common neurodegenerative diseases affecting over 6 million people worldwide.^[^ ^1^ ^]^ Classical motor symptoms of PD, such as resting tremor, bradykinesia, and rigidity, are primarily attributed to the progressive loss of dopaminergic (DA) neurons in the substantia nigra (SN).^[^ ^2^, ^3^ ^]^ Among the risk factors of PD, α‐synuclein (α‐Syn), encoded by the SNCA gene, is believed to play a key role in neuron death.^[^ ^4^ ^]^ Under physiological status, α‐Syn is highly enriched in the presynaptic terminals and mainly exists as soluble monomers.^[^ ^5^, ^6^ ^]^ When the balance between the production and clearance of α‐Syn is disturbed, the elevated α‐Syn is vulnerable to aggregate and misfold into protofibrils and finally Lewy body.^[^ ^7^, ^8^ ^]^ Misfolded α‐Syn exerts neurotoxicity and induces the onset of PD by promoting mitochondrial dysfunction, endoplasmic reticulum stress, synaptic impairment, and neuroinflammation. ^[^ ^9^, ^10^ ^]^Therefore, the defective turnover of α‐Syn is now considered as a pivotal pathogenic process in PD and offers several therapeutic targets for preventing DA neuron degeneration.

α‐Syn is cleared by lysosomal pathway and ubiquitin‐proteasome system, which maintains the intracellular homeostasis of α‐Syn.^[^ ^11^, ^12^, ^13^ ^]^ Many studies have shown that the way α‐Syn degradation was defined by different types of ubiquitin chains.^[^ ^14^, ^15^, ^16^, ^17^, ^18^ ^]^ Ubiquitination is a reversible process regulated by both E3 ligases and deubiquitinases (DUBs). E3 ligase neural precursor cell expressed developmentally downregulated 4 (NEDD4) promoted K63‐linked polyubiquitin chains of α‐Syn, leading to the lysosomal degradation via the endosomal‐sorting complex required for transport (ESCRT) pathway.^[^ ^19^ ^]^ Overexpression of E6 associated protein (E6‐AP) enhanced the proteasomal degradation of α‐Syn by increasing the ubiquitination of α‐Syn.^[^ ^20^ ^]^ The molecular chaperone and disaggregase activities of Tripartite motif 11 (TRIM11) cooperated with its SUMO ligase activity to degrade aberrant protein, by which TRIM11 abrogated α‐Syn fibrillization and mitigated α‐Syn‐mediated pathology in a PD mouse model.^[^ ^21^ ^]^ Meanwhile, some DUBs also participate in the degradation of α‐Syn. Ubiquitin specific proteinase 8 (USP8) inhibited the lysosomal degradation of α‐Syn by specifically hydrolyzing the K63‐linked polyubiquitin chains.^[^ ^22^ ^]^ Ubiquitin specific proteinase 9X (USP9X) knockdown promoted the accumulation of monoubiquitinated α‐Syn species and enhanced the formation of toxic α‐Syn inclusions upon proteolytic inhibition.^[^ ^23^ ^]^ Therefore, the molecular mechanisms by which DUBs regulate α‐Syn degradation are diverse and worthy of further study.

As a member of the ovarian tumor protease (OTU) family, OTUD5 has been implicated in diverse cellular processes and pathological conditions. OTUD5 was identified as a negative regulator of type I interferon (IFN‐I) by selectively cleaving K63‐linked polyubiquitin chains on tumor necrosis factor receptor‐associated factor 3.^[^ ^24^ ^]^ OTUD5 also played a role in acquired immunity by stabilizing UBR5 and inhibiting the production of IL‐17A in activated T cells.^[^ ^25^ ^]^ OTUD5 enhanced anti‐viral infection and anti‐tumor immunity by stabilizing STING.^[^ ^26^ ^]^ Recently, OTUD5 was reported to promote the inflammatory response by enhancing Myd88 oligomerization and Myddosome formation.^[^ ^27^ ^]^ Moreover, OTUD5 was shown to be involved in cancer development and progression.^[^ ^28^, ^29^, ^30^, ^31^ ^]^ In addition, OTUD5 functioned as a regulator of the DNA damage response by deubiquitinating Ku80^[^ ^32^ ^]^ or by stabilizing the UBR5.^[^ ^33^ ^]^ OTUD5 is expressed in various organs including the brain according to the Human Protein Atlas (HPA) (https://www.proteinatlas.org/) databases. RNA sequencing data from putamen and caudate also showed that expression of OTUD5 was decreased in PD patients,^[^ ^34^ ^]^ suggesting the possible relation between OTUD5 and PD. Therefore, we investigated whether OTUD5 is involved in the PD pathological process by regulating α‐Syn.

In the current study, we provided initial evidence that OTUD5 protected DA neurons by negatively regulating the protein levels of α‐Syn in the PD models both in vitro and in vivo. Mechanistically, OTUD5 acted as a scaffold to recruit the E3 ubiquitin ligase NEDD4, which promoted K63‐linked polyubiquitination and endolysosomal degradation of α‐Syn. Consistently, conditional knockout (CKO) of OTUD5 in DA neurons aggravated neurodegeneration induced by α‐Syn PFF in vivo. Taken together, our findings revealed a novel function of OTUD5 in regulating α‐Syn protein levels and provided new insights into the pathogenesis of PD.

## 2 Results

### 2.1 OTUD5 Specifically Facilitated the Degradation of a‐Syn

**Figure 1:**
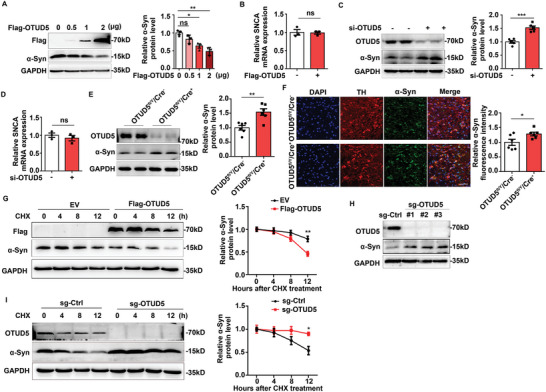
OTUD5 facilitated the degradation of a‐Syn. A) Gradient amount of Flag‐OTUD5 was transfected in SH‐SY5Y cells, the protein levels of α‐Syn were detected by western blot. Quantification of α‐Syn protein levels in SH‐SY5Y cells was normalized to GAPDH (n = 3 biologically independent). (B) RT‐PCR analysis of SNCA mRNA levels in SH‐SY5Y cells transfected with plasmid expressing OTUD5. Quantification of SNCA mRNA levels in SH‐SY5Y cells was normalized to β‐actin (n = 3 biologically independent). (C) Immunoblot analysis of α‐Syn protein level in SH‐SY5Y cells transfected with siRNA target for OTUD5. Quantification of α‐Syn protein levels in SH‐SY5Y cells was normalized to GAPDH (n = 5 biologically independent). (D) RT‐PCR analysis of SNCA mRNA levels in SH‐SY5Y cells transfected with siRNA targeting OTUD5. Quantification of SNCA mRNA levels in SH‐SY5Y cells was normalized to β‐actin (n = 3 biologically independent). (E) Immunoblot analysis and quantification of lysates from primary cultured midbrain neurons of WT or OTUD5 CKO mice. Quantification of α‐Syn protein levels was normalized to GAPDH (n = 6 biologically independent). (F) Immunofluorescence staining for α‐Syn (green) was performed in SN regions of OTUD5^fl/Y^/Cre^−^ and OTUD5^fl/Y^/Cre^+^ mice. Quantification of fluorescence intensity of α‐Syn was performed by ImageJ software (n = 6 mice per group). Scale bar, 20 µm. (G) Immunoblot analysis of extracts from SH‐SY5Y cells overexpressed OTUD5 treated with cycloheximide (CHX, 100 µg mL^−1^) (n = 3 biologically independent). The interaction between Flag‐OTUD5 and CHX treated time was statistically significant (P = 0.018), which was analyzed by repeated measure ANOVA. (H) Immunoblot analysis of extracts from SH‐SY5Y cells transfected with OTUD5 sgRNAs or scrambled sgRNA (sg‐Ctrl). (I) Immunoblot analysis of extracts from OTUD5 knockout SH‐SY5Y cells treated with CHX (n = 3 biologically independent). The interaction of sg‐OTUD5 and CHX treated time was statistically significant (P = 0.049), which was analyzed by repeated measure ANOVA. Data were expressed as the mean ± SEM. One‐way ANOVA followed by Tukey's post hoc test was used for statistical analysis in (A). Two‐tailed Student's t‐tests were used for statistical analysis in (B–F). Two‐way ANOVA followed by Tukey's post hoc tests were used for statistical analysis in (G) and (I). ns, not significant (*p* > 0.05), *p < 0.05, **p < 0.01, ***p < 0.001.

To identify the potential DUBs involved in α‐Syn regulation, we assessed α‐Syn protein levels after plasmids expressing various OTU family DUBs were transfected into SH‐SY5Y cells. A significant reduction in α‐Syn protein levels was observed upon OTUD5 overexpression (Figure S1, Supporting Information). To further validate the role of OTUD5 on α‐Syn regulation, OTUD5 was overexpressed or knockdown in SH‐SY5Y cells. As expected, OTUD5 overexpression remarkably diminished the protein levels of α‐Syn dose‐dependently in SH‐SY5Y cells (**Figure** **1**A), but no alteration was observed in its mRNA expression (Figure 1B). Consistently, knockdown of OTUD5 significantly enhanced α‐Syn protein expression in SH‐SY5Y cells (Figure 1C), while the mRNA levels remained unaffected (Figure 1D). Additionally, OTUD5 deficiency significantly enhanced α‐Syn protein levels in primary cultured midbrain neurons (Figure 1E). The upregulation of α‐Syn in DA neurons of OTUD5 CKO mice was confirmed by immunofluorescence (Figure 1F). As there are physiological functions for α‐Syn, we investigated whether OTUD5 overexpression and knockout can perturb the cellular steady state by regulating α‐Syn. The cell death and cell viability in SH‐SY5Y cells were assessed by propidium iodide (PI) staining and CCK8 respectively. The results showed that the rates of cell death and cell viability were not obviously affected by OTUD5 overexpression or knockout under normal state (Figure S2, Supporting Information). The data above indicated that OTUD5 negatively regulated α‐Syn protein level rather than the transcriptional level. To confirm this, we inhibited de novo protein synthesis by cycloheximide (CHX) and measured α‐Syn protein levels to evaluate the influence of OTUD5 on α‐Syn degradation. The results showed that OTUD5 overexpression facilitated the degradation of α‐Syn (Figure 1G). In addition, we designed and synthesized sgRNAs targeting human OTUD5 and verified the knockout efficiency in SH‐SY5Y cells by western blot. α‐Syn protein levels were also increased in SH‐SY5Y cells transfected OTUD5 sgRNAs (Figure 1H). Conversely, its degradation was attenuated in OTUD5 knockout SH‐SY5Y cells (Figure 1I). The data provided direct evidence that OTUD5 reduced the protein levels of α‐Syn by promoting its degradation.

### 2.2 OTUD5 Promoted Endolysosomal Degradation of α‐Syn

**Figure 2:**
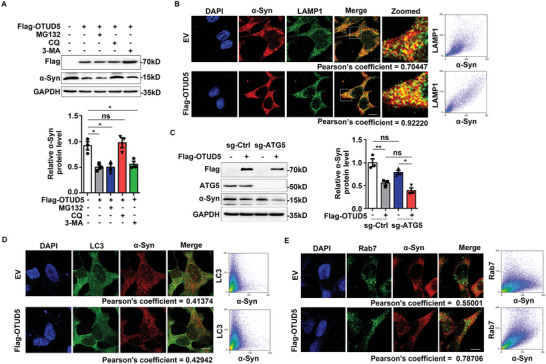
OTUD5 promoted endolysosomal degradation of α‐Syn. (A) Immunoblot analysis of extracts from SH‐SY5Y cells transfected with Flag‐OTUD5 then treated with MG132 (10 µM), chloroquine (CQ, 10 µM) or 3‐MA (10 mM) for 6 h. Quantification of α‐Syn levels in SH‐SY5Y cells was normalized to GAPDH (n = 3 biologically independent). (B) Representative confocal images of immunofluorescence staining for α‐Syn (red) and lysosome marker LAMP1 (green) in SH‐SY5Y cells transfected with the plasmids expressing Flag‐OTUD5 and followed by CQ treatment for 6 h. Scale bar, 10 µm. (C) Western blots were performed in lysates of ATG5 knockout (sg‐ATG5) cells and its control (sg‐Ctrl) cells transfected with Flag‐OTUD5. Quantification of α‐Syn protein levels was shown on the right (n = 3 biologically independent). The interaction of sg‐ATG5 and Flag‐OTUD5 was not statistically significant (P = 0.716), which was tested by 2*2 factorial analysis. (D) Confocal microscopic analysis of the co‐localization of endogenous α‐Syn (red) and LC3 (green). Scale bar, 10 µm. (E) Confocal microscopic analysis of the co‐localization of endogenous α‐Syn (red) and Rab7 (green). Scale bar, 10 µm. Co‐localization was quantified by using Pearson's correlation coefficient method and scatter map from ImageJ software in (B), (D), and (E). Data were expressed as the mean ± SEM. One‐way ANOVA followed by Tukey's post hoc test was used for statistical analysis in (A). Two‐way ANOVA followed by Tukey's post hoc test was used for statistical analysis in (C). ns, not significant (*p* > 0.05), *p < 0.05, **p < 0.01.

The ubiquitin‐proteasome pathway and lysosomal pathway are the two main systems involved in regulating α‐Syn degradation.^[^ ^12^, ^13^ ^]^ To determine the pathway by which OTUD5 induced the degradation of α‐Syn, we employed selective inhibitors and assessed the changes in endogenous α‐Syn levels. Proteasomal function was inhibited by MG132, chloroquine (CQ) was used to inhibit lysosomal function by preventing lysosomal acidification, and 3‐methyladenine (3‐MA) was used to inhibit autophagy by blocking autophagic vacuole formation. The concentrations were chosen to maximally inhibit these systems without causing any toxicity to cells.^[^ ^35^ ^]^ Interestingly, the degradation of endogenous α‐Syn induced by OTUD5 was evidently blocked by CQ, but not MG132 or 3‐MA (**Figure** **2**A). Furthermore, confocal microscopic analysis visualized that the co‐localization of α‐Syn and LAMP1, a marker of lysosomes, was significantly enhanced by OTUD5 overexpression (Figure 2B).

However, both autophagosomes and late endosomes can sequester cargo and finally fuse with lysosomes for protein degradation.^[^ ^36^ ^]^ Autophagy‐related proteins (ATGs) are key linkers in the autophagy pathway.^[^ ^37^ ^]^ Among them, autophagy related 5 (ATG5) is integral to both canonical and non‐canonical autophagy processes by initiating the formation of the autophagosome membrane and the fusion of autophagosomes and lysosomes.^[^ ^38^ ^]^ We utilized ATG5 knockout cells to investigate whether OTUD5 mediated the protein degradation of α‐Syn through the autophagic pathway. Our results showed that the degradation of α‐Syn induced by OTUD5 changed rarely in ATG5 knockout cells (Figure 2C). In addition, OTUD5 overexpression did not affect the co‐localization of endogenous α‐Syn and LC3 (Figure 2D). These data indicated that the degradation of α‐Syn induced by OTUD5 may not occur through autophagy‐lysosomal pathway. Since endosomes could also fuse with lysosomes to degrade protein, we next performed immunofluorescence staining of different endosome markers and α‐Syn. As anticipated, most α‐Syn co‐localized with Rab7‐positive late endosomes in SH‐SY5Y cells overexpressed OTUD5 (Figure 2E), while the co‐localization of α‐Syn and Rab5c‐positive early endosomes or Rab11a‐positive recycling endosomes barely changed (Figure S3A,B, Supporting Information). Collectively, these results provided evidence that OTUD5 promoted the transportation of α‐Syn to late endosomes and led to its degradation in lysosomes.

### 2.3 OTUD5 Interacted with α‐Syn and Promoted Its K63‐Linked Polyubiquitination

**Figure 3:**
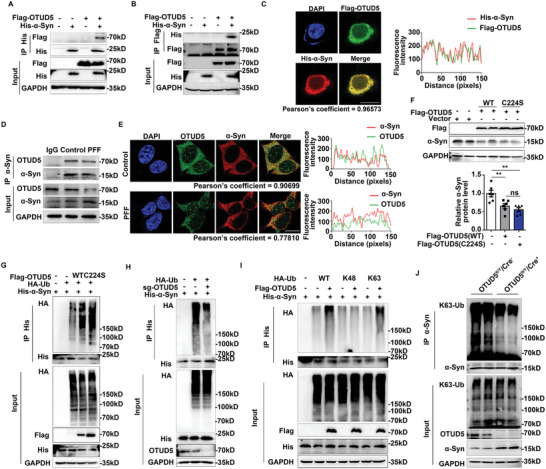
OTUD5 interacted with α‐Syn and promoted its K63‐linked ubiquitination. (A) Co‐immunoprecipitation (Co‐IP) was performed with lysates from HEK293T cells expressing His‐α‐Syn and Flag‐OTUD5. IP with anti‐His antibody probed with anti‐Flag antibody. (B) Co‐IP was performed with lysates from HEK293T cells expressing His‐α‐Syn and Flag‐OTUD5. IP with anti‐Flag antibody, probed with anti‐His antibody. (C) Confocal microscopy of HEK293T cells transfected with plasmids expressing Flag‐OTUD5 (green) and His‐α‐Syn (red). Scale bar, 10 µm. (D) Co‐IP was performed to analyze the endogenous OTUD5 and α‐Syn in SH‐SY5Y cells treated with α‐Syn PFF. (E) Confocal analysis of the co‐localization of endogenous α‐Syn (red) and OTUD5 (green) in SH‐SY5Y cells stimulated by α‐Syn PFF. Scale bar, 10 µm. (F) SH‐SY5Y cells transfected with Flag‐OTUD5 (WT) or Flag‐OTUD5 (C224S), α‐Syn protein level was detected by immunoblotting. Quantification of α‐Syn levels in SH‐SY5Y cells was normalized to GAPDH (n = 6 biologically independent). (G) Immunoblot analysis of lysates from HEK293T cells transfected with HA‐tagged ubiquitin (HA‐Ub), His‐α‐Syn, and Flag‐OTUD5 (WT) or Flag‐OTUD5 (C224S), followed by 6 h CQ (10 µM) treatment. IP with anti‐His antibody, probed with anti‐HA antibody. (H) Immunoblot analysis of lysates from HEK293T or OTUD5 knockout HEK293T cells transfected with HA‐tagged ubiquitin (HA‐Ub), His‐α‐Syn, followed by IP with anti‐His, probed with anti‐HA. (I) HEK293T cells were transfected with HA‐Ub, HA‐tagged K48‐linked ubiquitin (HA‐K48) or HA‐tagged K63‐linked ubiquitin (HA‐K63), His‐α‐Syn and Flag‐OTUD5 followed by 6 h CQ (10 µM) treatment. Cells were harvested, denatured, and lysed for immunoprecipitation with anti‐His antibody. The ubiquitination level of His‐α‐Syn was assessed by immunoblotting with anti‐HA antibody. (J) Co‐IP and immunoblot analysis of lysates from SN of OTUD5^fl/Y^/Cre^−^ and OTUD5^fl/Y^/Cre^+^ mice. IP with anti‐α‐Syn antibody, probed with anti‐K63‐Ub antibody. Co‐localization was quantified by using Pearson's correlation coefficient method. Intensity profiles of indicated proteins along the plotted lines were analyzed by ImageJ line scan analysis in (C) and (E). Data were expressed as the mean ± SEM. One‐way ANOVA followed by Tukey's post hoc test was used for statistical analysis in (F). ns, not significant (*p* > 0.05), **p < 0.01.

To further explore how OTUD5 promoted α‐Syn degradation, we examined whether OTUD5 interacted with α‐Syn. Firstly, Flag‐tagged OTUD5 and His‐tagged α‐Syn were expressed in HEK293T cells. Co‐immunoprecipitation (Co‐IP) assay clearly showed the interaction between OTUD5 and α‐Syn (**Figure** **3**A,B). The obvious co‐localization between exogenous OTUD5 and α‐Syn was observed in HEK293T cells by confocal analysis (Figure 3C). Besides, an association between endogenous OTUD5 and α‐Syn was evident in SH‐SY5Y cells (Figure 3D,E). OTUD5 is a 571‐amino‐acid protein containing a catalytic domain (OTU) from residues 220 to 340 and a short ubiquitin‐interacting motif (UIM) from residues 536 to 553.^[^ ^39^ ^]^ To detect which domain(s) of OTUD5 were responsible for the interaction with α‐Syn, three truncated mutants were constructed (Figure S4A, Supporting Information). Co‐IP results showed that 1–360 of OTUD5 mutant lost the ability to interact with α‐Syn, whereas OTUD5 mutant containing 220–571 residues has the ability to interact with α‐Syn. Therefore, 361–571 residues of OTUD5 are required for the interaction with α‐Syn (Figure S4B, Supporting Information).

Given that OTUD5 is a deubiquitinating enzyme, we investigated whether the OTUD5‐mediated degradation of α‐Syn depends on its enzymatic activity. We transfected plasmids encoding wild type (WT) OTUD5 or the enzymatic activity mutant OTUD5 (C224S) into SH‐SY5Y cells. The results showed that both WT OTUD5 and OTUD5 (C224S) led to a significant reduction in α‐Syn protein levels (Figure 3F). Next, we assessed whether OTUD5 modulated the ubiquitination levels of α‐Syn. Surprisingly, we observed that OTUD5 overexpression increased α‐Syn polyubiquitination (Figure 3G). Consistent with the above data that the mutant OTUD5 (C224S) promoted the degradation of α‐Syn, overexpression of OTUD5 (C224S) also dramatically enhanced the polyubiquitination levels of α‐Syn (Figure 3G). Meanwhile, OTUD5 knockout greatly decreased polyubiquitination of α‐Syn (Figure 3H). We then defined the type of polyubiquitin chains attached to α‐Syn mediated by OTUD5. HEK293T cells were transfected with the ubiquitin mutant K48 and K63, in which all of the lysine residues were replaced by arginine except for the one at position 48 and 63 respectively. OTUD5 overexpression significantly increased α‐Syn polyubiquitination in WT and K63 ubiquitin transfected cells but not in K48 ubiquitin transfected cells (Figure 3I). In addition, in order to exclude the possibility that HA‐signal is from α‐Syn interacting proteins, UREA‐lysis buffer was used to disrupt the interaction between proteins, and His‐tagged α‐Syn was purified by Ni‐NTA agarose beads.^[^ ^40^ ^]^ Consistently, the results further confirmed that OTUD5 promoted K63‐linked polyubiquitination on α‐Syn (Figure S5A,B, Supporting Information). Furthermore, we extracted proteins from SN of WT and OTUD5 CKO mice and assessed the endogenous ubiquitination of α‐Syn. As shown in Figure 3J, the K63‐linked polyubiquitination of α‐Syn was significantly attenuated in OTUD5 CKO mice compared with WT mice. Collectively, these data revealed that OTUD5 mediated K63‐linked polyubiquitination of α‐Syn independent of its enzymatic activity.

### 2.4 OTUD5 Recruited E3 Ligase NEDD4 to Promote the Degradation of α‐Syn

**Figure 4:**
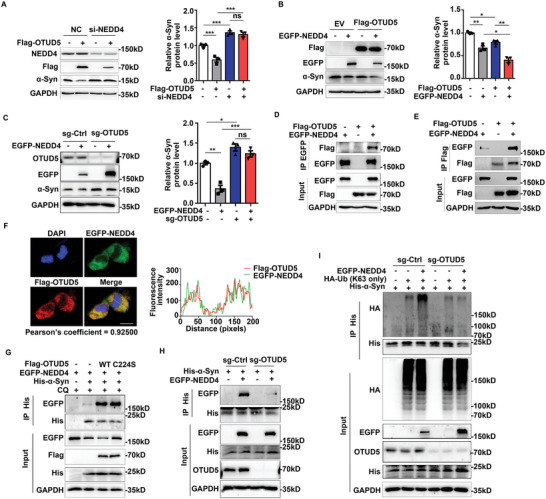
OTUD5 recruited E3 ligase NEDD4 to promote the degradation of α‐Syn. (A) SH‐SY5Y cells were transfected with siRNA for NEDD4 or negative control (NC) and Flag‐OTUD5 or the empty Flag‐vector (EV), the protein levels of α‐Syn were detected by western blot. Quantification of α‐Syn protein levels was shown on the right panel (n = 3 biologically independent). The interaction of si‐NEDD4 and Flag‐OTUD5 was significant (P = 0.012). (B) SH‐SY5Y cells were transfected with EGFP‐NEDD4 or empty EGFP‐vector and Flag‐OTUD5 or the empty Flag‐vector (EV), the protein levels of α‐Syn were detected by western blot. Quantification of intensity of α‐Syn was shown on the right panel (n = 3 biologically independent). The interaction of Flag‐OTUD5 and EGFP‐NEDD4 was not statistically significant (P = 0.509). (C) Overexpressed EGFP‐NEDD4 in SH‐SY5Y cells transfected sgRNA targeting OTUD5 (sg‐OTUD5) or sg‐Ctrl, the protein levels of α‐Syn were detected using an anti‐α‐Syn antibody. Quantification of the intensity of α‐Syn was shown on the right panel (n = 3 biologically independent). The interaction effect of sg‐OTUD5 and EGFP‐NEDD4 was statistically significant (P = 0.013). (D, E) Exogenous interactions between OTUD5 and NEDD4 were analyzed by Co‐IP in HEK293T cells transfected Flag‐OTUD5 and EGFP‐NEDD4. (F) Confocal microscopy of HEK293T cells transfected with EGFP‐NEDD4 (green) and Flag‐OTUD5 (red). Scale bar, 10 µm. (G) Co‐IP and immunoblot analysis of extracts of HEK293T cells transfected with EGFP‐NEDD4, His‐α‐Syn, and Flag‐OTUD5 or Flag‐OTUD5 C224S treated with CQ (10 µM) for 6 h. (H) The interaction between EGFP‐NEDD4 and His‐α‐Syn was attenuated in HEK293T OTUD5 knockout cells. (I) Co‐IP and immunoblot analysis of lysates from WT HEK293T cells or OTUD5 knockout HEK293T cells transfected with HA‐tagged K63‐linked ubiquitin (K63‐Ub), EGFP‐NEDD4 and His‐α‐Syn. IP with anti‐His antibody, probed with anti‐HA antibody. Co‐localization was quantified by Pearson's correlation coefficient method from ImageJ software. Intensity profiles of indicated proteins along the plotted lines were analyzed by ImageJ line scan analysis in (F). Data were expressed as the mean ± SEM. Two‐way ANOVA for 2*2 factorial analysis and Tukey's post hoc tests were used for statistical analysis in (A–C). ns, not significant (*p* > 0.05), *p < 0.05, **p < 0.01, ***p < 0.001.

Based on our finding that OTUD5 promoted α‐Syn polyubiquitination independent of its enzymatic activity, we hypothesized that OTUD5 may serve as an adaptor protein to recruit an E3 ligase for K63‐linked polyubiquitination and degradation of α‐Syn. Among the E3 ligases implicated in α‐Syn degradation, NEDD4 was reported to facilitate the endolysosomal degradation of α‐Syn by catalyzing its K63‐linked polyubiquitination.^[^ ^19^, ^41^ ^]^ Therefore, we investigated whether the degradation of α‐Syn mediated by OTUD5 relied on NEDD4. Firstly, we transfected siRNA specific to human NEDD4 into SH‐SY5Y cells and found that NEDD4 knockdown mitigated the degradation of α‐Syn induced by OTUD5 overexpression (**Figure** **4**A). NEDD4 overexpression further reduced the protein levels of α‐Syn compared to OTUD5 transfection alone (Figure 4B), while OTUD5 knockout impaired the degradation of α‐Syn induced by NEDD4 (Figure 4C). Previous studies reported that NEDD4 ubiquitinated cytosolic and aggregated α‐Syn.^[^ ^19^, ^42^ ^]^ Our double‐labeling immunofluorescence also showed that the co‐localization between OTUD5 and p‐α‐Syn (Ser 129), a marker of pathological aggregated α‐Syn, which was substantially enhanced upon α‐Syn PFF treatment in SH‐SY5Y cells, which indicated that OTUD5 may involve in the degradation of aggregated α‐Syn besides monomers (Figure S6, Supporting Information). Next, Co‐IP assays revealed that OTUD5 interacted with NEDD4 exogenously (Figure 4D,E), and 1–220 residues of OTUD5 were required for the interaction with NEDD4 (Figure S7, Supporting Information). Confocal microscopy further validated the interaction (Figure 4F). Interestingly, we found that OTUD5 overexpression enhanced the interaction between NEDD4 and α‐Syn (Figure 4G). Notably, OTUD5 (C224S) also promoted the association between NEDD4 and α‐Syn (Figure 4G), which confirmed that the deubiquitinating enzyme activity is not required. In contrast, OTUD5 knockout in HEK293T cells impaired the binding of NEDD4 and α‐Syn (Figure 4H). Finally, we demonstrated the K63‐linked polyubiquitination of α‐Syn induced by NEDD4 was significantly attenuated in OTUD5 knockout cells (Figure 4I). However, the overexpression or absence of OTUD5 did not affect the protein levels of NEDD4 obviously and vice versa (Figure S8A‐D, Supporting Information). Overall, these results indicated that OTUD5 recruited NEDD4 to promote K63‐linked polyubiquitination of α‐Syn and subsequent degradation.

### 2.5 OTUD5 Was Downregulated in α‐Syn PFF‐induced PD Model

**Figure 5:**
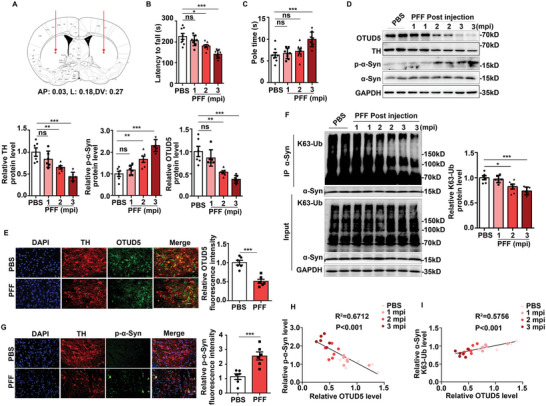
OTUD5 was downregulated in PD mouse model induced by α‐Syn PFF. Wild type (WT) mice were injected in bilateral striatum (STR) with α‐Syn PFF (5µg each side) or PBS at 1, 2, and 3 months post injection (mpi). (A) Schematic diagram of stereotactic injection of PBS or α‐Syn PFF in the STR. (B) Rotarod test and (C) pole test were used to measure motor coordination (n = 8 mice per group). (D) Immunoblot analysis of protein expression levels in SN from WT mice injected with α‐Syn PFF at 1, 2, and 3 mpi. Quantification of indicated protein levels was normalized to GAPDH (n = 6 mice per group). (E) Photomicrographs and quantification of OTUD5 (green) and TH (red) in SN sections of mice after α‐Syn PFF treatment (n = 6 mice per group). Scale bars, 20 µm. (F) Co‐IP and immunoblot analysis of SN lysates from WT mice injected with α‐Syn PFF at 1, 2, and 3 mpi. IP with anti‐α‐Syn antibody, probed with anti‐K63‐Ub antibody. Quantification of indicated protein levels was normalized to GAPDH (n = 6 mice per group). (G) Photomicrographs and quantification of SN sections stained for p‐α‐Syn (Ser129) (green), TH (red), and DAPI from mice injected with α‐Syn PFF at 3 mpi (n = 6 mice per group). Scale bar, 20 µm. (H) Pearson's correlation analysis showing the correlations between OTUD5 and p‐α‐Syn levels in mice brain tissues (n = 6 mice per group; n = 24 mice in total). (I) Pearson's correlation analysis showing the correlation between OTUD5 and K63‐linked polyubiquitylation of α‐Syn in mice brain tissues (n = 6 mice per group; n = 24 mice in total). Data were expressed as the mean ± SEM. One‐way ANOVA followed by Tukey's test was used for statistical analysis in (B‐D, F). Two‐tailed Student's t‐tests were used for statistical analysis in (E) and (G). ns, not significant (*p* > 0.05), *p < 0.05, **p < 0.01, ***p < 0.001.

OTUD5 is expressed in various organs including brain (https://www.proteinatlas.org/). We also verified that OTUD5 was distributed in the cortex, hippocampus, SN, and striatum (STR) (Figure S9, Supporting Information). To explore the contribution of OTUD5 to the development of PD, we performed stereotaxic injection of α‐Syn PFF to mice which is a well‐recognized way to induce PD mouse model ^[^ ^43^, ^44^, ^45^, ^46^, ^47^ ^]^ (**Figure** **5**A). First, the neuron toxicity caused by α‐Syn PFF injection was assessed at different time points. Motor coordination was assessed using the rotarod and pole test. WT mice injected with α‐Syn PFF at 2 and 3 months post injection (mpi) exhibited a shorter latency to fall from the rod compared to control mice (Figure 5B). In the pole test, the time that WT mice spent on the pole was prolonged at 3 months after α‐Syn PFF injection (Figure 5C). Furthermore, the protein levels of tyrosine hydroxylase (TH) were decreased time‐dependently after injection of α‐Syn PFF (Figure 5D), demonstrating the pathological manifestation in this model was evident at this time point. Immunohistochemistry (IHC) staining further confirmed the loss of DA neurons in the SN and STR of mice injected with α‐Syn PFF at 3 mpi (Figure S10A,B, Supporting Information). Notably, α‐Syn PFF injection significantly induced the downregulation of OTUD5 protein levels in a time‐dependent manner, with the lowest levels at 3 mpi (Figure 5D). Besides, double‐immunofluorescence staining showed that OTUD5 in DA neurons of SN was significantly downregulated after α‐Syn PFF injection (Figure 5E). Meanwhile, we found that the majority of OTUD5‐positive cells were NeuN‐positive neurons, while a smaller proportion of OTUD5‐positive cells were GFAP‐positive astrocytes or Iba1‐positive microglia (Figure S11A–C, Supporting Information). Consistently, OTUD5 was downregulated both in SH‐SY5Y cells and primary cultured midbrain neurons treated by α‐Syn PFF (Figure S11D–F, Supporting Information). Moreover, we checked the mRNA level of OTUD5 in PD mice models. Consistent with the data from patients,^[^ ^34^ ^]^ the mRNA levels of OTUD5 were also decreased obviously in SN of mice injected with α‐Syn PFF (Figure S12A, Supporting Information) and in SH‐SY5Y cells stimulated by α‐Syn PFF (Figure S12B, Supporting Information). These data indicated that OTUD5 expression was reduced at the transcriptional level possibly. Coherently, the endogenous K63‐linked polyubiquitination levels of α‐Syn in SN were also reduced significantly after α‐Syn PFF injection (Figure 5F). In addition, p‐α‐Syn (Ser129) was obviously detected in the SN of mice injected with α‐Syn PFF (Figure 5D,G). Next, Pearson's analyses were conducted to check the relation between OTUD5 and the PD pathological process. Of note, OTUD5 was negatively correlated with p‐α‐Syn (Ser 129) (Figure 5H), while it positively correlated with K63‐linked polyubiquitylation of α‐Syn in PD mouse model induced by α‐Syn PFF (Figure 5I). Collectively, these findings shed insight on the involvement of OTUD5 in PD by regulating K63‐linked polyubiquitination of α‐Syn.

### 2.6 OTUD5 Conditional Deficiency Compromised Dopaminergic Neurodegeneration in PD Mouse Model Induced by α‐Syn PFF

**Figure 6:**
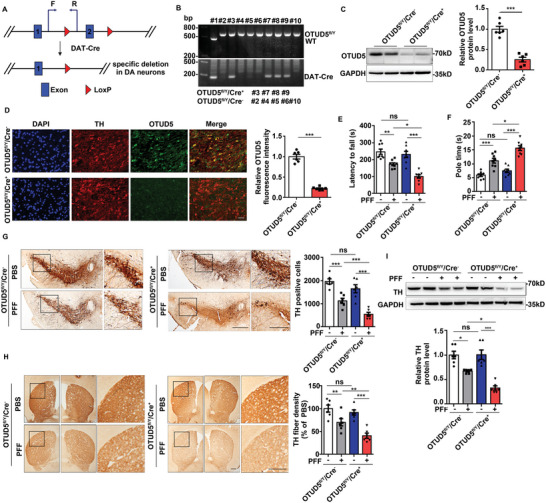
OTUD5 conditional deficiency compromised dopaminergic neurodegeneration in PD mouse model induced by α‐Syn PFF. (A) A diagram for the mice generation of OTUD5 specific deletion in dopaminergic (DA) neurons. (B) PCR was performed on genomic DNA extracted from mice tail to confirm mice genotypes. (C) Western blot analysis of lysates from SN of OTUD5^fl/Y^/Cre^−^ and OTUD5^fl/Y^/Cre^+^ mice. Quantification of indicated protein levels was shown on the right (n = 6 mice per group). (D) Immunofluorescence staining for TH (red) and OTUD5 (green) was performed in SN regions of OTUD5^fl/Y^/Cre^−^ and OTUD5^fl/Y^/Cre^+^ mice. Quantification of fluorescence intensity of OTUD5 were performed by ImageJ software (n = 6 mice per group). Scale bar, 20 µm. (E) Rotarod test and (F) pole test were used to evaluate the motor coordination on mice injected with α‐Syn PFF at 3 mpi (n = 8 mice per group). The interaction of OTUD5 conditional knockout (CKO) and α‐Syn PFF was statistically significant in (E) (P = 0.046). In (F), the interaction of OTUD5 CKO and α‐Syn PFF was significant (P = 0.033). (G) Micrographs of DA neurons in SN of OTUD5^fl/Y^/Cre^−^ and OTUD5^fl/Y^/Cre^+^ mice treated with PBS or α‐Syn PFF by TH staining. Scale bar, 200 µm. Quantification of TH‐positive neurons in the SN was measured by ImageJ software (n = 6 mice per group). The interaction of OTUD5 CKO and α‐Syn PFF was not statistically significant (P = 0.293). (H) Micrographs of STR sections stained for TH fiber density of OTUD5^fl/Y^/Cre^−^ and OTUD5^fl/Y^/Cre^+^ mice treated with PBS or α‐Syn PFF. Scale bar, 200 µm. The average optical density of DA fiber in the STR was measured by ImageJ software and the data were normalized to WT‐PBS group (n = 6 mice per group). The interaction between OTUD5 CKO and α‐Syn PFF was not statistically significant (P = 0.145). (I) Immunoblot analysis of lysates from SN of OTUD5^fl/Y^/Cre^−^ and OTUD5^fl/Y^/Cre^+^ mice treated with PBS or α‐Syn PFF. Quantification of indicated protein levels was normalized to GAPDH (n = 6 mice per group). The interaction of OTUD5 CKO and α‐Syn PFF was statistically significant (P = 0.015). Data were expressed as the mean ± SEM. Two‐tailed Student's t‐tests were used for statistical analysis in (C) and (D). Two‐way ANOVA for 2*2 factorial analysis and Tukey's post hoc tests were used for statistical analysis in (E‐I). *p < 0.05, **p < 0.01, ***p < 0.001.

To elucidate the essential role of OTUD5 in DA neurodegeneration, we generated mice with targeted deletion of OTUD5 specifically in DA neurons using the recombinase Cre: loxP system by crossing OTUD5‐floxed mice with transgenic mice expressing Cre recombinase under the dopamine transporter (DAT) promoter^[^ ^48^ ^]^ (**Figure** **6**A). In the resulting strain, conditional deletion of OTUD5 in DA neurons occurred only in Cre‐expressing floxed mice (OTUD5^fl/Y^/Cre^+^, #3 #7 # 8 #9) and not in mice lacking Cre expression floxed mice (OTUD5^fl/Y^/Cre^−^, #2 #4 #5 #6 #10) (Figure 6B). The efficiency of deletion was also confirmed by western blot analysis and immunofluorescent staining in the SN (Figure 6C,D). In order to reveal the function of OTUD5 in PD, 10‐week‐old OTUD5 CKO mice and their OTUD5^fl/Y^/Cre^−^ littermates were stereotaxically injected with α‐Syn PFF for 3 months. Motor behavior was evaluated by the rotarod and pole test. As shown in Figure 6E,F, the deletion of OTUD5 in DA neurons significantly aggravated the α‐Syn PFF‐induced behavioral deficits. In addition, IHC staining showed that the loss of DA neurons was obviously increased in OTUD5 deficient mice compared to WT mice treated with α‐Syn PFF, which was assessed by the number of TH‐positive neurons in the SN (Figure 6G) and the density of TH‐positive fibers in the STR (Figure 6H). Western blot analysis confirmed that the reduction of TH protein levels was further enhanced in SN of OTUD5 CKO mice after α‐Syn PFF injection (Figure 6I). Similarly, the absence of OTUD5 also exacerbated the reduction of TH in primary cultured midbrain neurons treated with α‐Syn PFF (Figure S13A Supporting Information). In contrast, a pronounced reduction in TH levels induced by α‐Syn PFF was rescued by OTUD5 overexpression in SH‐SY5Y cells (Figure S13B Supporting Information).

**Figure 7:**
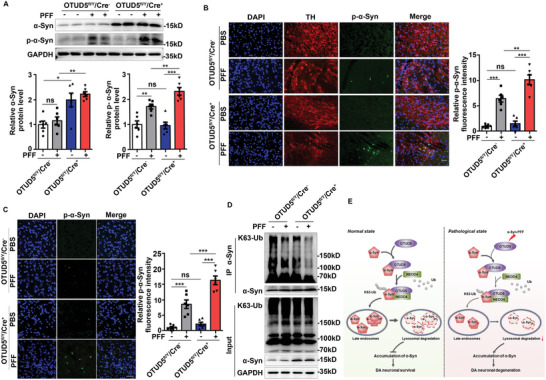
OTUD5 protected dopaminergic neurons in α‐Syn PFF‐induced PD model. (A) Immunoblot analysis of lysates from SN of OTUD5^fl/Y^/Cre^−^ and OTUD5^fl/Y^/Cre^+^ mice treated with PBS or α‐Syn PFF. Quantification of indicated protein levels was normalized to GAPDH (n = 6 mice per group). The interaction between OTUD5 CKO and α‐Syn PFF was not statistically significant in regulating α‐Syn level (P = 0.831) while the interaction was significant in regulating p‐α‐Syn (Ser129) level (P = 0.036). (B) Representative micrographs of SN sections stained for p‐α‐Syn (Ser129) (green) and TH (red) of OTUD5^fl/Y^/Cre^−^ and OTUD5^fl/Y^/Cre^+^ mice treated with PBS or α‐Syn PFF. Scale bar, 20 µm. Quantification of p‐α‐Syn (Ser129) immunofluorescence intensity was shown on the right panel (n = 6 mice per group). The interaction between OTUD5 CKO and α‐Syn PFF was statistically significant (P = 0.004). (C) Representative micrographs of STR regions stained for p‐α‐Syn (Ser129) (green) of OTUD5^fl/Y^/Cre^−^ and OTUD5^fl/Y^/Cre^+^ mice treated with PBS or α‐Syn PFF. Quantification of p‐α‐Syn (Ser129) immunofluorescence intensity was performed by ImageJ software (n = 6 mice per group). Scale bar, 20 µm. The interaction between OTUD5 CKO and α‐Syn PFF was statistically significant (P = 0.003). (D) Co‐IP and immunoblot analysis of SN lysates from OTUD5^fl/Y^/Cre^−^ and OTUD5^fl/Y^/Cre^+^ mice treated with PBS or α‐Syn PFF. IP with anti‐α‐Syn antibody, probed with anti‐K63‐Ub antibody. (E) Schematic illustration for OTUD5 negatively regulating α‐Syn protein. Under normal state, the constitutively expressed OTUD5 recruited NEDD4 to α‐Syn and promoted the K63‐linked polyubiquitination of α‐Syn, leading to its degradation through the endolysosomal pathway. Thus, the balance between the generation and degradation of α‐Syn maintained DA neuronal homeostasis (left panel). In the pathological state of PD induced by α‐Syn PFF, the downregulated OTUD5 in DA neurons attenuated the interaction between NEDD4 and α‐Syn and the following endolysosomal degradation of α‐Syn, leading to the elevated protein level of α‐Syn. The upregulated α‐Syn accelerated the conversion into pathological α‐Syn and DA neuron loss induced by α‐Syn PFF (right panel). Data were expressed as the mean ± SEM. Two‐way ANOVA for 2 × 2 factorial analysis and Tukey's post hoc tests were used for statistical analysis. *p < 0.05, **p < 0.01, ***p < 0.001.

The protein levels of α‐Syn were obviously increased in SN of OTUD5 CKO mice. Notably, OTUD5 conditional deletion further increased pathological p‐α‐Syn induced by α‐Syn PFF (**Figure** **7**A). Immunofluorescence also showed that OTUD5 deficiency upregulated the formation of pathologic p‐α‐Syn (Ser129) in TH‐positive neurons in SN (Figure 7B) and STR (Figure 7C). Consistently, the fluorescence intensity of p‐α‐Syn (Ser129) was further enhanced in primary cultured midbrain neurons from OTUD5 CKO mice after α‐Syn PFF stimulation (Figure S14A, Supporting Information). Moreover, immunoblot analysis also revealed that depletion of OTUD5 further upregulated p‐α‐Syn protein levels in α‐Syn PFF‐treated primary cultured midbrain neurons (Figure S14B, Supporting Information). Furthermore, OTUD5 deficiency further attenuated the endogenous K63‐linked polyubiquitination of α‐Syn induced by α‐Syn PFF (Figure 7D). Jointly, the findings mentioned above suggested the protective role of OTUD5 on α‐Syn PFF‐exposed DA neurons by promoting the K63‐linked polyubiquitination and degradation of α‐Syn (Figure 7E).

## 3 Conclusion and Discussion

The abnormal clearance and accumulation of α‐Syn is the key pathogenic factor in PD, which leads to the loss of DA neurons and behavioral deficits.^[^ ^49^ ^]^ Thus, a reduction in α‐Syn could be a potential therapeutic approach for PD. Here, we found a crucial function of OTUD5 in negatively regulating the protein levels of α‐Syn. Particularly, OTUD5 selectively promoted K63‐linked polyubiquitination of α‐Syn and mediated its endolysosomal degradation by recruiting the E3 ligase NEDD4. In addition, genetic deletion of OTUD5 in DA neurons significantly aggravated the α‐Syn PFF‐induced neurodegeneration in vivo, while OTUD5 overexpression rescued the DA neuron damage induced by α‐Syn PFF in vitro.

Recent studies emphasized the importance of DUBs in regulating the fate of α‐Syn.^[^ ^50^ ^]^ USP8 hydrolyzed the K63‐linked polyubiquitin chains of α‐Syn and prevented its lysosomal degradation.^[^ ^22^ ^]^ USP9X co‐localized with α‐Syn and maintained the proteostasis by removing the monoubiquitin from α‐Syn.^[^ ^23^ ^]^ OTUD5 has been found to play important roles in multiple cellular signal pathways, including immunity,^[^ ^24^, ^25^, ^26^ ^]^ cancer progression,^[^ ^28^, ^29^, ^30^, ^31^ ^]^ and DNA damage.^[^ ^32^, ^33^ ^]^ However, whether OTUD5 is involved in PD pathogenesis by regulating α‐Syn degradation has not been studied. Here, we revealed that OTUD5 facilitated α‐Syn degradation through lysosomal pathway as the degradation was blocked by CQ, not MG132 or 3‐MA. Furthermore, OTUD5 promoted the transportation of α‐Syn to late‐endosomes rather than autophagosomes, indicating that OTUD5 regulated the degradation of α‐Syn by the endolysosomal pathway.

Unlike the function of USP8 and USP9X in regulating α‐Syn,^[^ ^22^, ^23^ ^]^ our result showed that OTUD5 facilitated K63‐linked polyubiquitin chains on α‐Syn and promoted its degradation. It has been noticed that DUBs have broad and diverse working patterns that are dependent or independent of their enzyme activities.^[^ ^51^ ^]^ DUBs can enhance the ubiquitination of target proteins besides cleaving the ubiquitin chains from the substrates. Ubiquitin C‐terminal hydrolase L1 (UCH‐L1) ubiquitinated α‐Syn and was linked to higher susceptibility to PD by its dimerization‐dependent ligase activity.^[^ ^52^ ^]^ USP18 functioned as a scaffold protein to facilitate the interaction between TRIM31 and MAVS, and promoted K63‐linked polyubiquitination and subsequent aggregation of MAVS.^[^ ^53^ ^]^ USP5 promoted K48‐linked polyubiquitination of NLRP3 and mediated its degradation in autophagy by recruiting E3 ligase MARCHF7.^[^ ^54^ ^]^ Interestingly, in our report, OTUD5 enhanced, but not cleaved, the K63‐linked ubiquitination levels of α‐Syn and degraded it independent of its deubiquitinase activity. These inspired us that OTUD5 may act as a critical adaptor protein and recruit a specific E3 ligase to regulate the degradation of α‐Syn.

So far, NEDD4 is the only known E3 ligase that could directly catalyze K63‐linked polyubiquitination on α‐Syn and mediate its degradation through the endolysosomal pathway.^[^ ^19^ ^]^ NEDD4 overexpression protected against α‐Syn accumulation and toxicity by decreasing the cytosolic and aggregated α‐Syn.^[^ ^42^ ^]^ To validate our hypothesis that OTUD5 may recruit NEDD4 to degrade α‐Syn, we first demonstrated that OTUD5 failed to enhance the degradation of α‐Syn when NEDD4 was knockdown, while NEDD4 overexpression promoted the degradation of α‐Syn induced by OTUD5, which indicated that OTUD5 mediated α‐Syn degradation relying on NEDD4. Second, an interaction between OTUD5 and NEDD4 was indeed detected. Besides, the residues 1–220 in N‐terminal region of OTUD5 were required for the interaction with NEDD4. Third, knockout of OTUD5 greatly inhibited the interaction between NEDD4 and α‐Syn, and decreased the degradation and K63‐linked polyubiquitination of α‐Syn catalyzed by NEDD4, which indicated that NEDD4‐mediated polyubiquitination and degradation of α‐Syn depended on OTUD5. Taken together, our findings demonstrated OTUD5 acted as a key scaffold protein recruiting E3 ligase NEDD4 to α‐Syn, and promoting K63‐linked polyubiquitination followed by endolysosomal degradation of α‐Syn.

Bilateral striatal injection of α‐Syn PFF was used to induce PD mouse model in this study.^[^ ^55^ ^]^ Our result showed that OTUD5 was located in DA neurons, and the protein levels of OTUD5 were obviously reduced in PD models induced by α‐Syn PFF. Moreover, we observed that the mRNA levels of OTUD5 were also decreased, which was consistent with RNA sequencing data from PD patients,^[^ ^34^ ^]^ These data suggested that the downregulation of OTUD5 in PD may be due to the reduced transcription. The detailed mechanism deserves further research. We then confirmed the function of OTUD5 in PD models. In α‐Syn PFF model, exogenous α‐Syn fibrils recruited endogenous soluble α‐Syn protein, accelerating the accumulation of α‐Syn and formation of phosphorylated pathological species.^[^ ^56^, ^57^ ^]^ Compared with WT mice, conditional deletion of OTUD5 caused further loss of TH‐positive neurons, accompanied with severer motor deficits. Besides, overexpressing OTUD5 restored the protein levels of TH in α‐Syn PFF‐induced PD cell model. Mechanically, we found that OTUD5 deficiency further reduced endogenous K63‐linked polyubiquitination of α‐Syn and upregulated its protein level, then promoted the formation of pathologic p‐α‐Syn and aggravated the pathogenic consequences induced by α‐Syn PFF.

In summary, we demonstrated that OTUD5 negatively regulated α‐Syn protein levels by promoting endolysosomal degradation independent of its deubiquitinate activity. Our study suggested a complicated regulatory mechanism of α‐Syn degradation, emphasizing its supreme importance in α‐Syn pathology. It identified OTUD5 as a novel therapeutic target and provided a promising therapeutic strategy for PD.

## 4 Experimental Section

### Cell Lines

Human Embryonic Kidney 293T (HEK293T) cells and Human neuroblastoma SH‐SY5Y cells were cultured in 1 × Dulbecco's modified Eagle's medium (Meilunbio, MA0212), supplemented with 10% fetal bovine serum (LONSERA, S711‐001S), penicillin (100 U/ml) (Meilunbio, M0110). All cells were maintained in a humidified incubator at 37 °C, in a 5% CO2 atmosphere.

### Reagents

MG132 (HY‐13259) and 3‐MA (HY‐19312) were purchased from MedChemExpress. CHX (S7418) and CQ (S6999) were purchased from Selleck.

### Plasmids, siRNAs, and Transfection

Plasmids His‐α‐Syn and EGFP‐NEDD4 were purchased from MIAOLING BIOLOGY. Flag‐OTUs and other plasmids were kept in our laboratory. siRNAs were synthesized and purified by GenePharma. The transient silencing target sequences are listed in Table S1 (Supporting Information). Plasmids or siRNAs were transfected into HEK293T cells by Polyethylenimine HCl MAX, MW 40 000(PEI MAX) (Polysciences, 24 765) according to the manufacturer's instructions. SH‐SY5Y cells were transfected using Lipofectamine 2000 reagents (Thermo, 11 668 019) according to the manufacturer's instructions. For IP experiments, we seeded 1200000 HEK 293T cells on 60 mm cell culture dishes and used 2 µg of every kind of plasmid or 4 µg siRNA for transfection. Scrambled siRNAs were consistently used as a negative control in siRNA experiments. In ubiquitination experiments, we transfected 3 µg His‐α‐Syn, 2 µg Flag‐OTUD5 or its mutant and 1 µg HA‐ubiquitin. As for experiments to investigate the protein regulation, we seeded 700000 SH‐SY5Y cells on 6‐well cell culture plates and transfected 1.5 µg of every kind of plasmid or 2 µg siRNA. The plasmids were transfected for 36 h and siRNAs were transfected for 48 h before collecting.

### Generation of Knockout HEK293T and SH‐SY5Y Cells

Knockout cells were generated according to the protocol described previously.^[^ ^58^ ^]^ Firstly, sgRNAs target OTUD5 and ATG5 were designed based on the http://www.genome‐engineering.org. The sgRNAs sequences were listed on Table S1 (Supporting Information). Secondly, the sgRNA was cloned into a lenti‐CRISPRV2 vector, and then the cloned product was transformed into E. coli DH5α competent cells. Thirdly, the successful plasmids were transiently transfected with plasmid encoding PSP‐2 and VSV‐G into HEK293T cells. The virus supernatant was harvested after 48 h transfection. Fourth, HEK293T or SH‐SY5Y cells were transduced with lentivirus at 50∼60% confluency. 48 h later, the cells were selected by media supplemented with Puromycin (Solarbio, P8230) 2µg/ml for HEK293T cells and 4µg/ml for SH‐SY5H cells respectively. Finally, protein levels were assessed by western blot.

### Western Blot

Brain areas that exhibit neuropathology including the SN and STR of mice were dissected and stored at −80 °C. The step‐by‐step description of dissection was performed as described previously and a tutorial film could also be found.^[^ ^59^ ^]^ Mouse brain tissues and cultured cells were homogenized and prepared in RIPA lysis buffer (Meilunbio, P0013B) containing a protease inhibitor (Beyotime, ST506) and Phosphatase Inhibitor Cocktail (Selleck, B15001) on ice. Protein concentrations were determined using a BCA protein assay reagent kit (Meilunbio, MA0082). In IP experiments, 500–1000 µg protein was loaded on the western blot gels for IP fraction, 20–40 µg protein was loaded for input fraction. The densitometry was quantified by using ImageJ software. The following primary antibodies were used: ATG5 (Cell Signaling Technology, 12 994; 1:1000), α‐Syn phospho S129) (abcam, ab51253; 1:500;), α‐Syn (BD biosciences, 610 786; 1:500), HA (OriGene Technologies, TA180128; 1:2000), Flag (Sigma Aldrich, F1804; 1:2000), His (Proteintech, 66005‐1; 1:1000), K63‐linkage specific polyubiquitin (Cell Signaling Technology, 5621S; 1:1000), OTUD5 (Proteintech, 21002‐1‐AP; 1:1000), NEDD4 (Proteintech, 21698‐1‐AP; 1:1000), GAPDH (Proteintech, 60004‐1; 1:30000), GFP (Santa Cruz Biotechnology, sc‐9996; 1:1000) and TH (Proteintech, 25859‐1‐AP; 1:2000). Secondary antibodies: Goat Anti‐Rabbit IgG(H+L) (Jackson ImmunoResearch, 111‐035‐144; 1:20000), Goat Anti‐Mouse IgG(H+L) (Jackson ImmunoResearch,115‐035‐003; 1:20000), HRP‐Protein A (Proteintech, SA00001‐18;1:2000).

### RT‐PCR Analysis

Total RNA was isolated from cultured cells using RNA‐Quick purifaction Kit (ES Science, RN001) according to the manufacturer's protocol. 2 µg of total RNA were reverse‐transcribed into cDNA using HiScript II Q RT SuperMix for qPCR (+gDNA wiper) (Vazyme, R223‐01) following the manufacturer's protocol. Quantitative PCR (real‐time PCR) was performed in triplicate using UltraSYBR Mixture (CWBIO, CW2624), and a Bio‐Rad iCycler system (Bio‐Rad Laboratories, Hercules, USA) with Bio‐Rad CFX Manager 2.1 software was used to analyze the mRNA levels for target genes. Levels of the housekeeping gene β‐actin were used as an internal control. The primers for the RT‐PCRs are included in Table S1 (Supporting Information).

### Co‐IP

In brief, total protein was extracted with IP‐buffer (150 mM NaCl, 1% NP‐40, 10 mM Tris‐Hcl, and 1 mM EDTA) containing a protease inhibitor (Beyotime, ST506‐2) and Phosphatase Inhibitor Cocktail (Selleck, B15001) on ice. Then the cell lysates were centrifuged at 13 800 xg at 4 °C for 10 min and the supernatants were collected and incubated with the indicated antibodies and corresponding IgG controls (Santa Cruz Biotechnology, sc‐2025) at 4 °C for 4 h. Next, Protein A&G magnetic beads for IP (Selleck, B23202) were mixed overnight at 4 °C under rotation. Finally, the beads were washed and eluted in SDS sample buffer for further analysis. The data were collected by SageCapture (MiniChemi610, Beijing, China).

### α‐Syn Ubiquitination Assay

Cells were lysed in a UREA‐lysis buffer (8M urea, 100 mM Na2HPO4/NaH2PO4, pH 8.0, 10 mM Tris‐HCl (pH 8.0), 5 mM imidazole, 10 mM β‐mercaptoethonal, and 1× protease inhibitor cocktail). Then the cell lysates were subjected to brief sonication before spinning down at 12000 rpm for 10 min. The supernatants were incubated with Ni‐NTA agarose beads (Qiagen, 30 210) for 6 h at room temperature with gentle agitation. The beads were then washed four times for 5 min each as follows: first with UREA lysis buffer; second with UREA wash buffer (8M UREA, 100 mM Na2HPO4/NaH2PO4, 10 mM Tris‐HCl (pH 6.8), 5 mM imidazole, 10 mM β‐mercaptoethonal); third with UREA‐wash buffer + 0.1% Triton X‐100; last with PBS. After washing, the His‐α‐Syn was eluted with elution buffer (0.2 M imidazole, 0.15 M Tris‐HCl (pH 6.8), 30% glycerol, 0.72 M β‐mercaptoethonal, 5% SDS). The eluates were then subjected to western blotting.

### Immunofluorescence

HEK293T cells, SH‐SY5Y cells or primary cultured neurons were seeded on glass coverslips in 6‐well plates and transfected with specific plasmids. The cells were fixed in 4% paraformaldehyde (Sigma, P6148) for 15 min at room temperature, rinsed with PBS three times (5 min/times), permeabilized with 0.5% BSA contained 0.2% Triton X‐100 for 20 min at room temperature, and blocked with 10% BSA for 1 h at room temperature. The cells were rinsed with PBS, and then stained with the indicated primary antibodies overnight at 4 °C, followed by incubation with fluorescent‐dye‐conjugated secondary antibodies. The nuclei were redyeing with DAPI (Beyotime, C1005). Brain sections were permeabilized with 0.5% BSA containing 0.4% Triton X‐100 for 20 min at room temperature, and blocked with 10% BSA for 1 h at room temperature. The following primary antibodies were used: Iba1 (Proteintech, 10904‐1‐AP; 1:100), GFAP (Millipore, MAB360; 1:200), NeuN (Millipore, MAB377; 1:200), TH (Millipore, MAB318; 1:400), α‐Syn (phospho S129) (abcam, ab51253; 1:100), α‐Syn (phospho S129) (abcam, ab184674; 1:100), α‐Syn (BD biosciences, 610 786; 1:100), α‐Syn (Immunoway biotechnology, YT5731; 1:100), OTUD5 (abcam, ab176727; 1:100), Rab5c (Abways Technology, CY10629; 1:200), Rab7 (Abways Technology, CY5814; 1:200), Rab11a (Abways Technology, CY5301; 1:200), FLAG (OriGene Technologies, TA592569; 1:200), His (Proteintech, 66005‐1; 1:200), LAMP1(Proteintech, 21997‐1‐AP; 1:200), LC3(Cell Signaling Technology, 2775;1:200), β‐III‐Tublin (Cell Signaling Technology,4466S; 1:200). Secondary antibodies: CoraLite488‐conjugate Goat Anti‐Rabbit IgG (Proteintech, SA00013‐2; 1:200), CoraLite594‐conjugated Goat Anti‐Mouse IgG (Proteintech, SA00013‐3; 1:200).Images were acquired with 400× magnification, 630× magnification (LSM980 Zeiss, Oberkochen, Germany) or 200× magnification (Olympus BX53, Tokyo, Japan). The co‐localization was measured with ImageJ software.

### Mice

Wild type male C57BL/6 mice were obtained from the Jinan Pengyue Experimental Animal Breeding Co., Ltd. OTUD5^fl/Y^ mice were C57BL/6 genetic background and generated by the Nanjing Biomedical Research Institute of Nanjing University (Nanjing, China) and provided by Dr Lingqiang Zhang, State Key Laboratory of Proteomics, Beijing Proteome Research Center, Beijing Institute of Radiation Medicine. Approximately 1.1 kb of the genomic region covering exon 2 was removed via Cre/loxP excision. Deletion of exon 2 caused a frameshift that disrupted downstream protein domains. DAT‐Cre transgenic mice were C57BL/6 genetic background and generated by Cyagen Biosciences Inc. (C001454, Suzhou, China). OTUD5^fl/Y^ mice were bred with DAT‐Cre transgenic mice to produce OTUD5 conditional knockout mice. Mouse genotyping was performed using genomic DNA isolated from mouse tails by PCR. The specific primers in this study are listed in Table S1 (Supporting Information). Age‐matched OTUD5^fl/Y^/Cre^−^ littermates were used as wild type controls in indicated experiments. All animal experiments were undertaken in accordance with the NIH Guide for the Care and Use of Laboratory Animals and received the approval of the Ethical Committee of the School of Basic Medical Sciences, Shandong University (No. ECSBMSSDU2020‐2‐103).

### Primary Neuronal Culture

The primary cultured midbrain neurons were prepared as previously described. ^[^ ^60^ ^]^ In brief, sacrificed pregnant mice and collected E13.5 embryos in dishes and washed them with D‐Hanks buffer (Meilunbio, MA0039). Under a dissection microscope, dissected ventral mesencephalon from the embryos were placed in tubes, and a small piece of tissue was cut from the embryos for genotyping. Each embryo corresponded strictly to the tube. Added Trypsin‐EDTA (Gibco Life Technologies, 25 200 072) to the dissected brain fragments and digested at 37 °C for 15 min. Then removed trypsin by centrifugation, added serum containing‐culture medium and performed mechanical cell dissociation with a pipet (10 trituration movements, repeated twice). Cells were plated on 6‐well plates at a density of 1000 000 cells/well or Poly‐D‐Lysine Hydrobromide (Gibco Life Technologies, A38904‐01)‐coated glass coverslips in 6‐well plates at a density of 600 000 cells/well. Cells were maintained in Neurobasal medium (Gibco Life Technologies, A8904‐01) supplemented with B27 (Gibco Life Technologies, 17504‐044) and GlutaMax (Gibco Life Technologies, 35050–061).

### Using of α‐Syn PFF In Vitro and In Vivo

Active Mouse Recombinant α‐Syn PFF (GTX17671‐pro) was purchased from GeneTex. For experiments in vitro, α‐Syn PFF was added to cell culture media to a final concentration of 5 µg mL^−1^ for 72 h. For stereotaxic injection of α‐Syn PFF, 10‐week‐old male mice were anesthetized with 0.5% sodium pentobarbital, striatal coordinates for stereotactic injection were anteroposterior (AP) +0.3 mm, mediolateral (ML) ±1.85 mm, dorsoventral (DV) +2.7 mm. The infusion was performed at a rate of 0.4 µL min^−1^, and 2.5 µL of α‐Syn PFF (2 µg µL^−1^) or the same volume of PBS was injected into each side of mouse brain.

### Rotarod Test

For the rotarod test, mice were placed on an accelerating rotarod cylinder, and the time the animals remained on the rotarod was measured. The speed was slowly increased from 4 to 40 rpm within 490 s. A trial ended if the animal fell off the rotarod or gripped the device and spun around for two consecutive revolutions without attempting to walk on the rotarod. The animals were trained 3 days before the test. Motor test data were presented as the mean duration (three trials) on the rotarod.

### Pole Test

Mice were acclimatized in the behavioral procedure room for 20 min. The pole was made of a 50‐cm metal rod with a diameter of 1 cm. It was wrapped with bandage gauze. Mice were placed on the top of the pole. The total time taken to reach the base of the pole was recorded. Before the actual test, mice were trained for two consecutive days. Each training session consisted of three test trials. On the test day, mice were evaluated in three trials and the total time was recorded. The maximum cutoff time to stop the test and recording was 60 s. Data were presented as the meantime (three trials) of time to climb down.

### Immunohistochemistry and Quantitative Analysis

Immunohistochemistry was performed on 20‐µm thick serial free floating brain sections. Free floating sections were first incubated in methyl alcohol containing 3% hydrogen peroxide for 10 min to block internal peroxidase activity, subsequently permeabilized with 0.2% Triton X‐100 for 15 min, and blocked with 10% fetal bovine serum (FBS) for 30 min at room temperature. Specimens were then incubated for 24 h at 4 °C with mouse monoclonal anti‐TH antibody (Millipore, MAB318; 1:200) in 2% BSA. After several washes with PBS, sections were incubated with Reagent 2 and Reagent 3 at 37 °C for 20 min respectively according to the manufacturer's instructions (ZSGB‐Bio, PV‐9000). Immuno‐complexes were revealed by 3, 3′‐diamino‐benzidine (ZSGB‐Bio, ZLI‐9018). Images were acquired with the Olympus BX53 (Olympus, Tokyo, Japan) microscope at 40 × or 100 × magnification with cellSens software (Olympus, Tokyo, Japan).

Quantification of TH positive neurons in SN and the density of dopaminergic terminals in STR were performed in a blinded manner according to the protocol described previously.^[^ ^44^, ^61^, ^62^ ^]^ For SN sections, every 7th section from the starting of SN (around 2.8 mm from bregma) was considered for counting of TH neurons. In detail, images were captured at the same exposure time and analyzed by ImageJ. Threshold was selected under Image/Adjust in order to achieve a desired range of intensity values to show a single neuron for each experiment. Once determined, this threshold was applied to all the images in each experiment. The selected area in the signal intensity range of the threshold was measured by the Analyze Particles. For STR sections, the average optical density of the whole STR was measured using the ImageJ software, and the staining signal was calibrated by subtracting the baseline signal of the cortex. The data of average optical density were normalized to the WT mice injected with PBS group.

### Statistics

Statistics were performed using GraphPad Prism 6 and SPSS 26. Unpaired two‐tailed Student's t tests, one‐way or two‐way ANOVA was used to assess the statistical significance. Data were presented as mean ± SEM. The number of replicates and repeats of individual experiments and statistical analysis was indicated in each figure legend. *p < 0.05, **p < 0.01 and ***p< 0.001 were considered significant.

## Conflict of Interest

The authors declare no conflict of interest.

## Supporting information



Supporting Information

## Data Availability

The data that support the findings of this study are available from the corresponding author upon reasonable request.
