# Supplementary material for: OTUD5 Protects Dopaminergic Neurons by Promoting the Degradation of α‐Synuclein in Parkinson's Disease Model
Source: Adv Sci (Weinh). 2024 Dec 25;12(7):2406700. doi: 10.1002/advs.202406700 (PMC11831440; doi:10.1002/advs.202406700)
Supplement: Supplementary file 1 — Supporting Information [file ADVS-12-2406700-s001.docx]

Supporting Information

**OTUD5 protects dopaminergic neurons by promoting the degradation of α-synuclein in Parkinson’s disease model**

*Xiaomeng Song#, Tengfei Liu#, Lu Yu, Qiuran Ji, Xin Guo, Runzhe Zong, Yiquan Li, Gan Huang, Qidi Xue, Qingyi Fu, Bingyu Liu, Yi Zheng, Lin Chen, Chengjiang Gao*, Huiqing Liu**


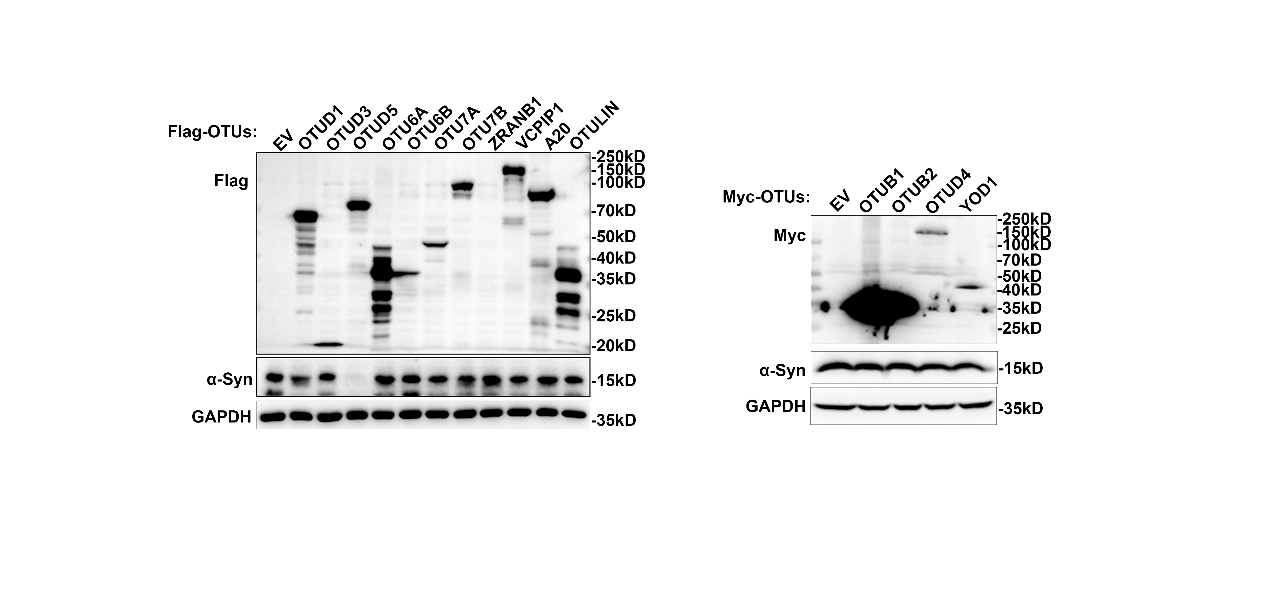


**Figure S1.** Screening of OTU family DUBs regulating α-Syn protein level. The plasmids expressing indicated DUBs were transfected into SH-SY5Y cells. Cell lysates were subjected to western blotting analysis with indicated antibodies.


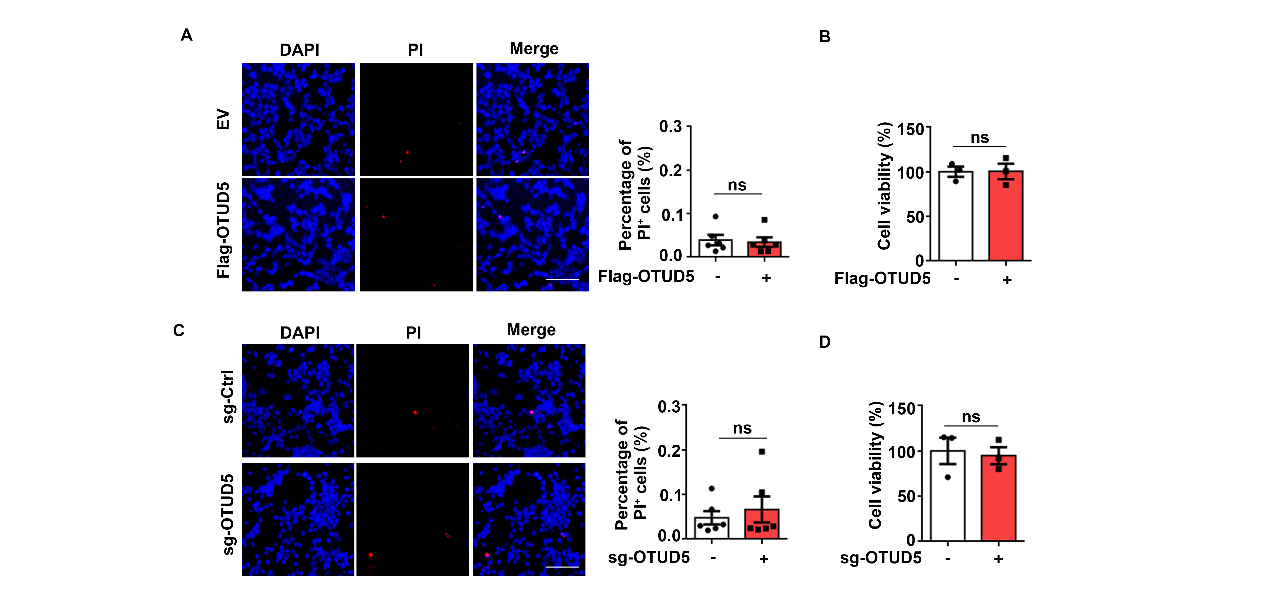


**Figure S2.** The effect of OTUD5 on cell death and cell viability in SH-SY5Y cells. (A)The propidium iodide (PI) staining for SH-SY5Y cells transfected with Flag-OTUD5 plasmids or empty vector (EV) (n = 6 biologically independent experiments). (B) The cell viability was detected by CCK-8 assay in SH-SY5Y cells transfected with Flag-OTUD5 plasmids or EV (n = 3 biologically independent experiments). (C) PI staining for SH-SY5Y cells transfected with OTUD5 sgRNA or scrambled sgRNA (sg-Ctrl) (n = 6 biologically independent experiments). (D) CCK-8 assay was performed to detect the cell viability of SH-SY5Y cells transfected with OTUD5 sgRNA or scrambled sgRNA (sg-Ctrl) (n = 3 biologically independent experiments). Data were expressed as the mean ± SEM. ns, not significant (P > 0.05). Two-tailed Student’s t-tests were used for statistical analysis.


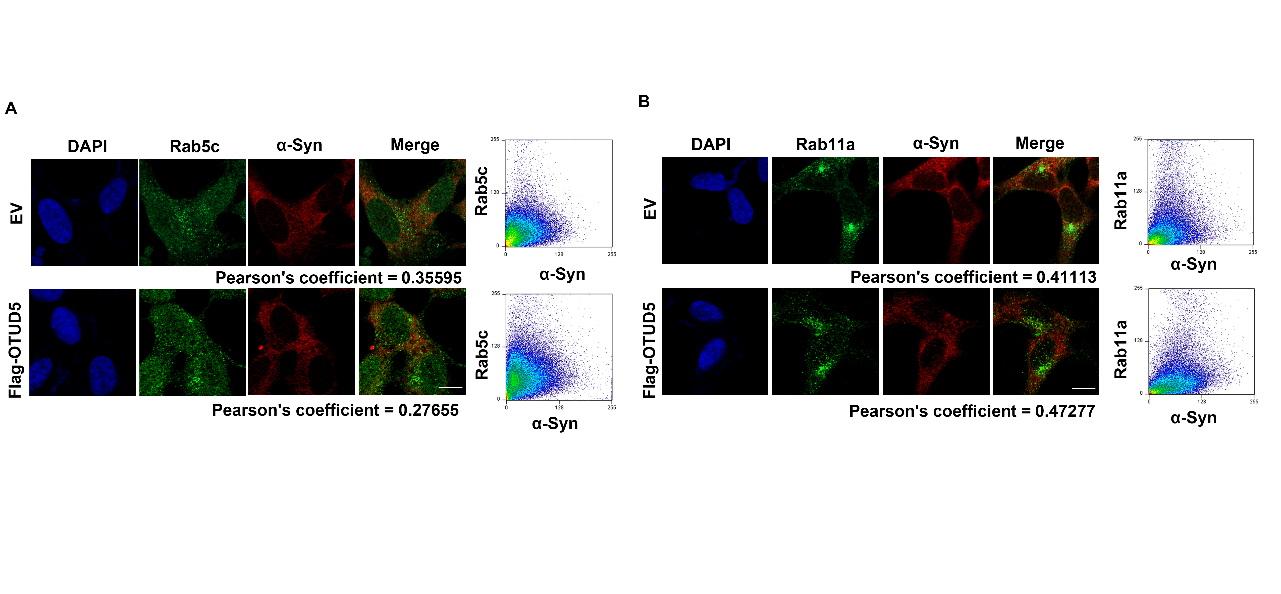


**Figure S3.** Co-localization of α-Syn and early endosomes or recycling endosomes was not affected by OTUD5 overexpression. The plasmids expressing Flag-OTUD5 or EV were transfected into SH-SY5Y cells and followed by CQ (10 µM) treatment for 6 h. Confocal analysis of the co-localization of endogenous α-Syn (red) and Rab5c (green) (A) or Rab11a (green) (B). Scale bar, 10 μm. Co-localization was quantified by using Pearson’s correlation coefficient method from Image J software.

**
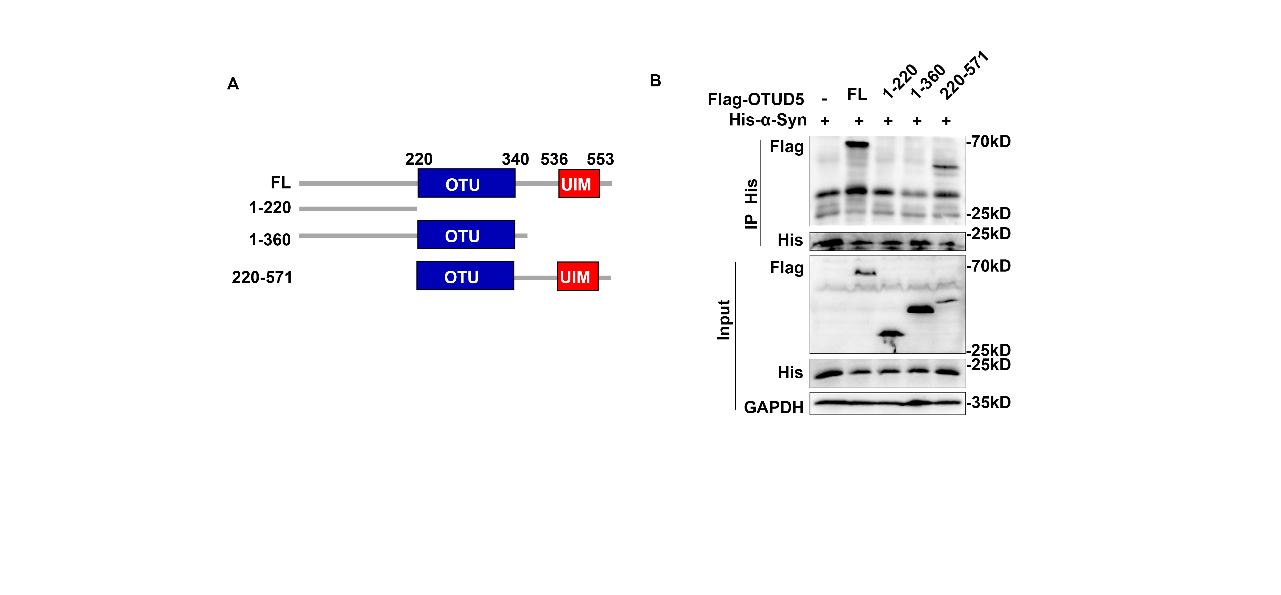
**

**Figure S4.** Residues from 361-571 of OTUD5 were responsible for interaction with α-Syn. (A) Schematic diagram of OTUD5 and its truncation mutants. (B) Flag-OTUD5 or its mutants and His-α-Syn were individually transfected into HEK293T cells. The cell lysates were immunoprecipitated with an anti-His antibody and then immunoblotted with the indicated antibodies.


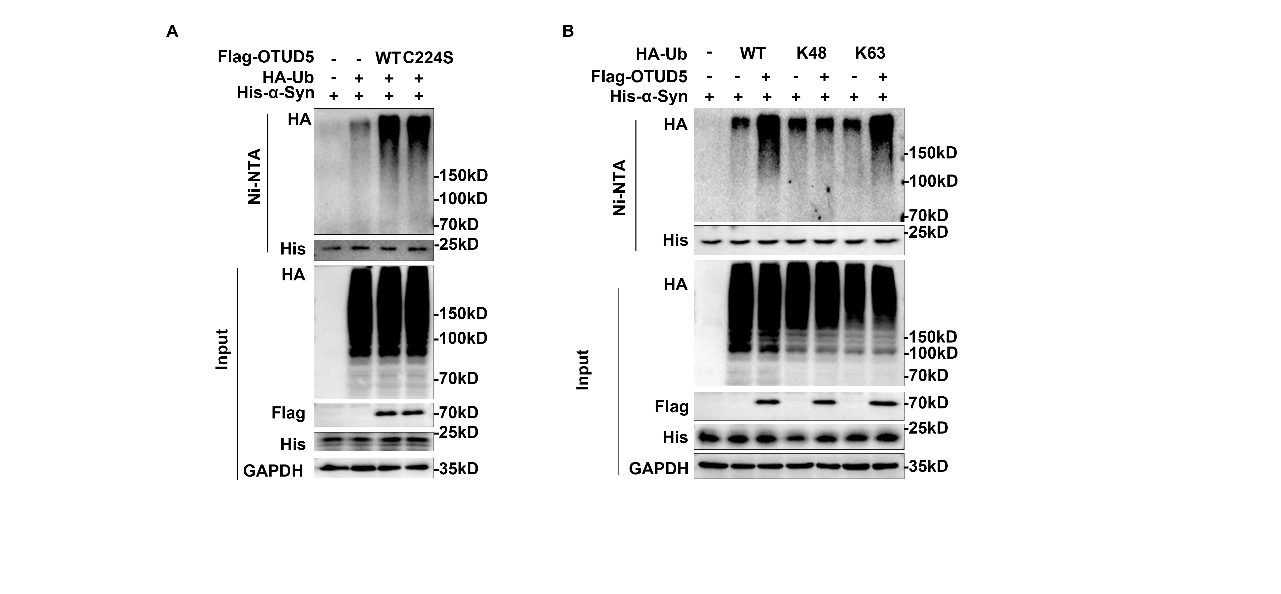


**Figure S5.** OTUD5 promoted the K63-linked polyubiquitination of α-Syn. (A) HEK293T cells were transfected with plasmids expressing His-α-Syn and HA-Ub, as well as a control vector or plasmids expressing Flag-OTUD5 (WT) or Flag-OTUD5 (C224S). (B) HEK293T cells were transfected with plasmids expressing His-α-Syn, Flag-OTUD5, HA-Ub and its mutants that can only append either K48 or K63 ubiquitin chains. The cells were lysed in 8M UREA-containing lysis buffer and then subjected to His-tag pulldown by Ni-NTA agarose beads. The α-Syn precipitates were then probed with anti-HA antibody on western blot.


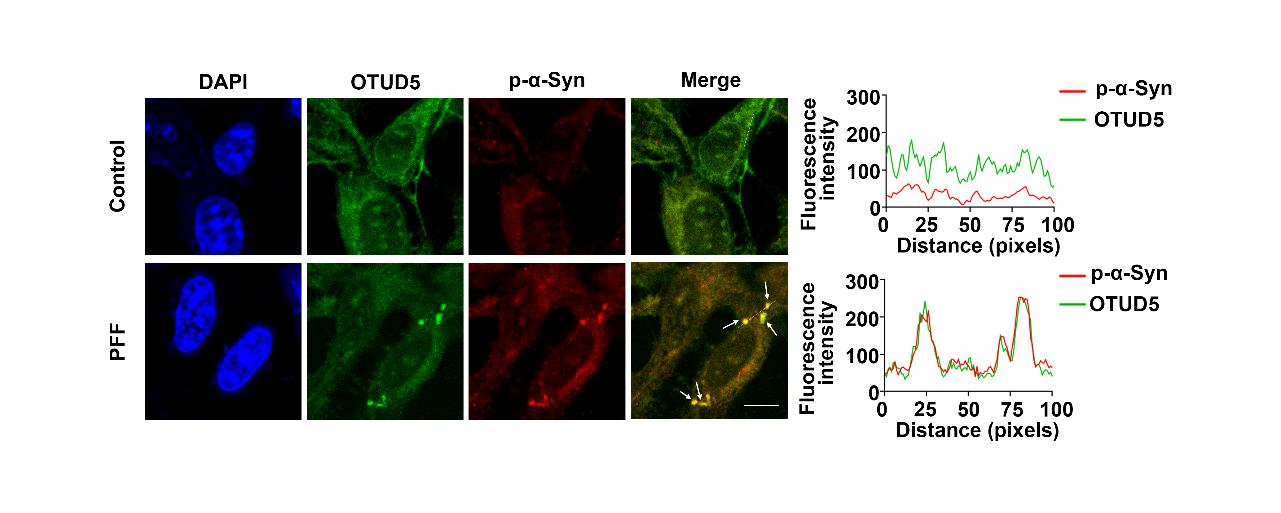


**Figure S6.** OTUD5 co-localized with p-α-Syn (Ser 129) in SH-SY5Y cells treated with α-Syn PFF. Confocal microscopic analysis of the co-localization of endogenous p-α-Syn (Ser 129) (red) and OTUD5 (green). Intensity profiles of indicated proteins along the plotted lines were analyzed by ImageJ line scan analysis. Scale bar, 10 μm. The white arrows indicated the detected co-localization of p-α-Syn and OTUD5.

**
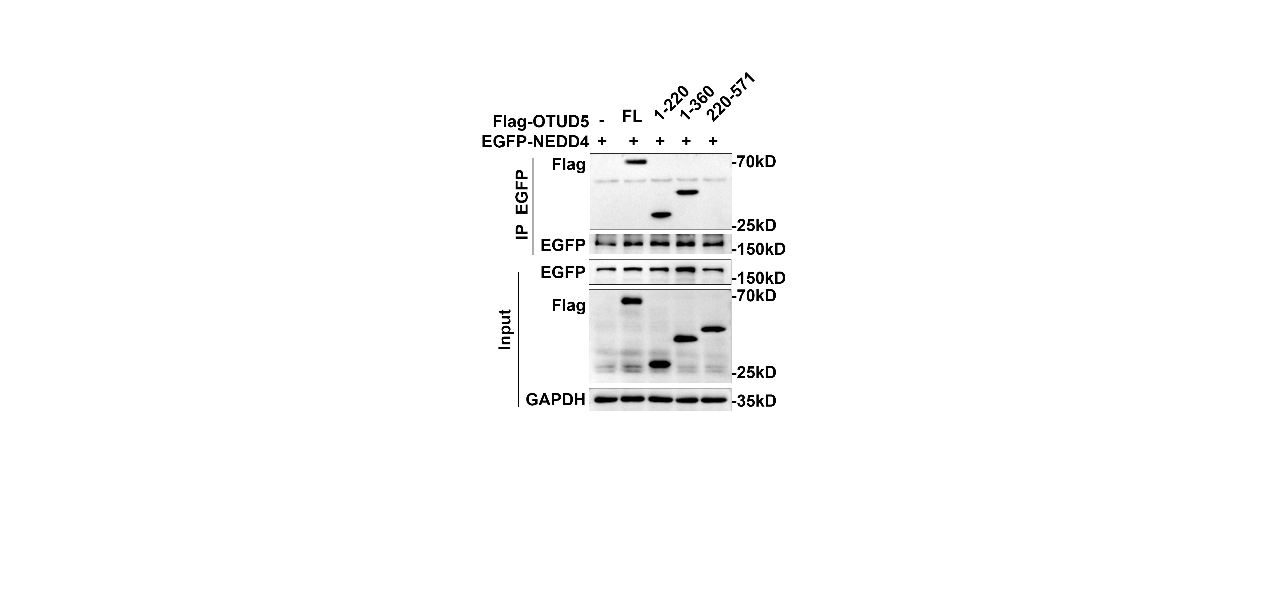
**

**Figure S7.** Residues from 1-220 of OTUD5 were required for the interaction with NEDD4. Flag-OTUD5 or its truncated mutants and EGFP-NEDD4 were individually transfected into HEK293T cells. The cell lysates were immunoprecipitated with an anti-EGFP antibody and then immunoblotted with the indicated antibodies.


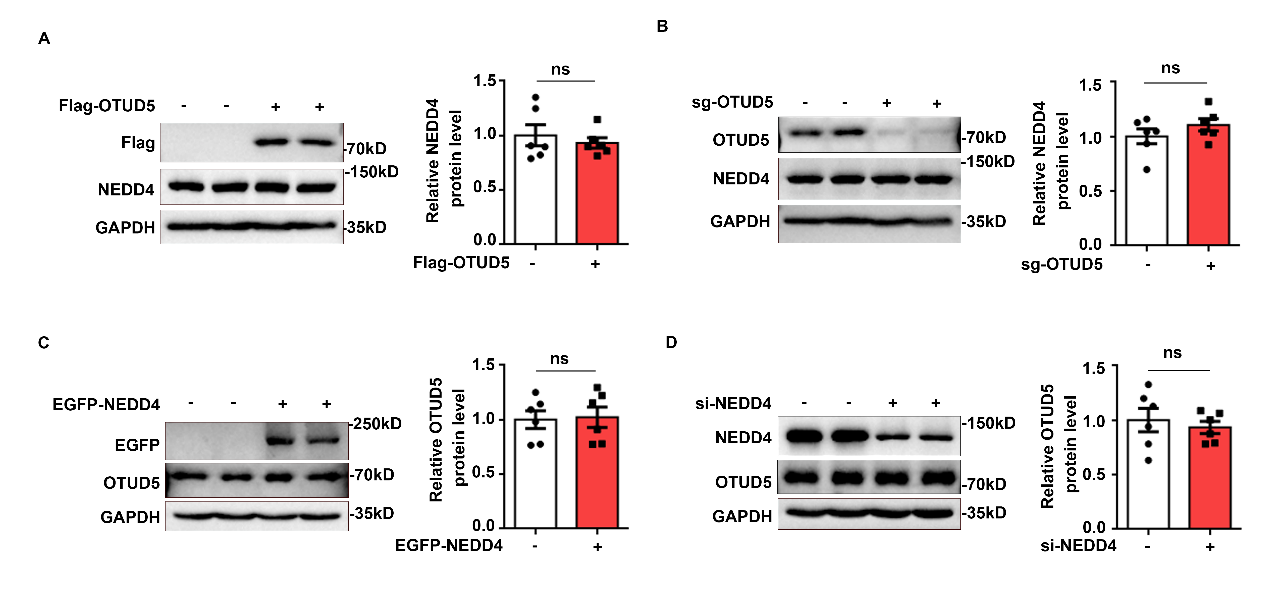


**Figure S8.** OTUD5 and NEDD4 barely affected the expression of each other. (A) The protein levels of NEDD4 were detected by western blot in SH-SY5Y cells overexpressed OTUD5. (B) The protein levels of NEDD4 were detected by western blot in OTUD5 knockout SH-SY5Y cells. (C)The protein levels of OTUD5 were detected by western blot in SH-SY5Y cells overexpressed NEDD4. (D) The protein levels of OTUD5 were detected by western blot in SH-SY5Y cells transfected by si-NEDD4. n = 6 biologically independent. Data were expressed as the mean ± SEM. Two-tailed Student’s t-tests were used for statistical analysis. ns, not significant (P > 0.05).


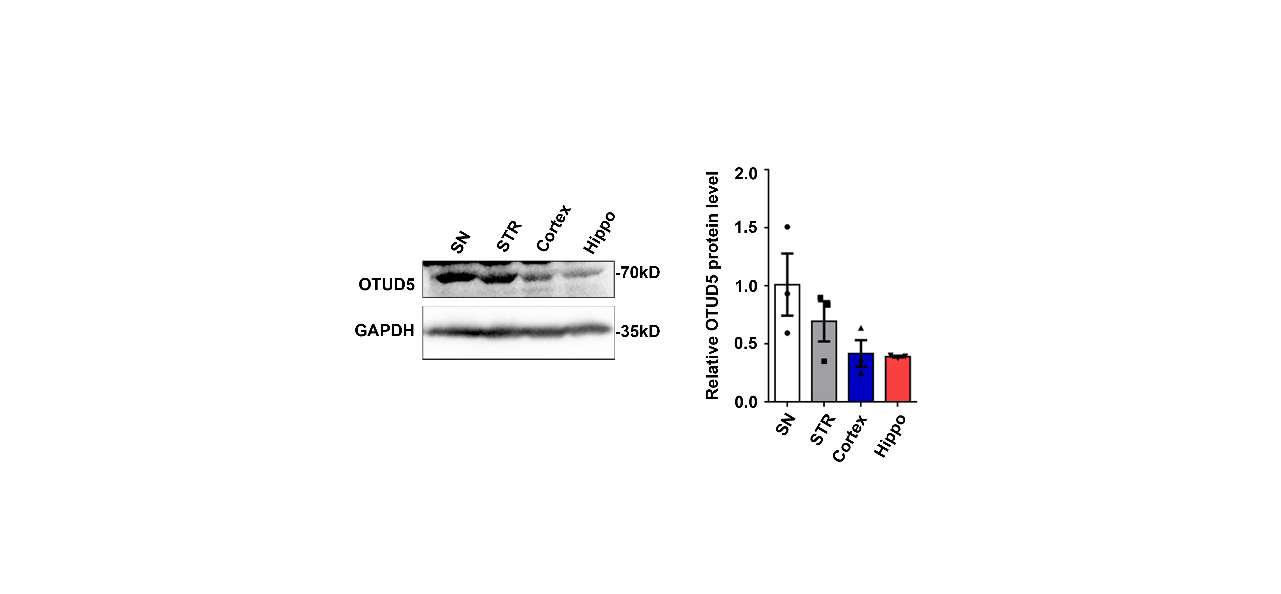


**Figure S9.** OTUD5 was expressed in different encephalic regions. The protein levels of OTUD5 in SN, STR, Cortex and Hippocampus (Hippo) in WT mice. Quantification of OTUD5 levels were normalized to GAPDH (n = 3 biologically independent). Data were expressed as the mean ± SEM.


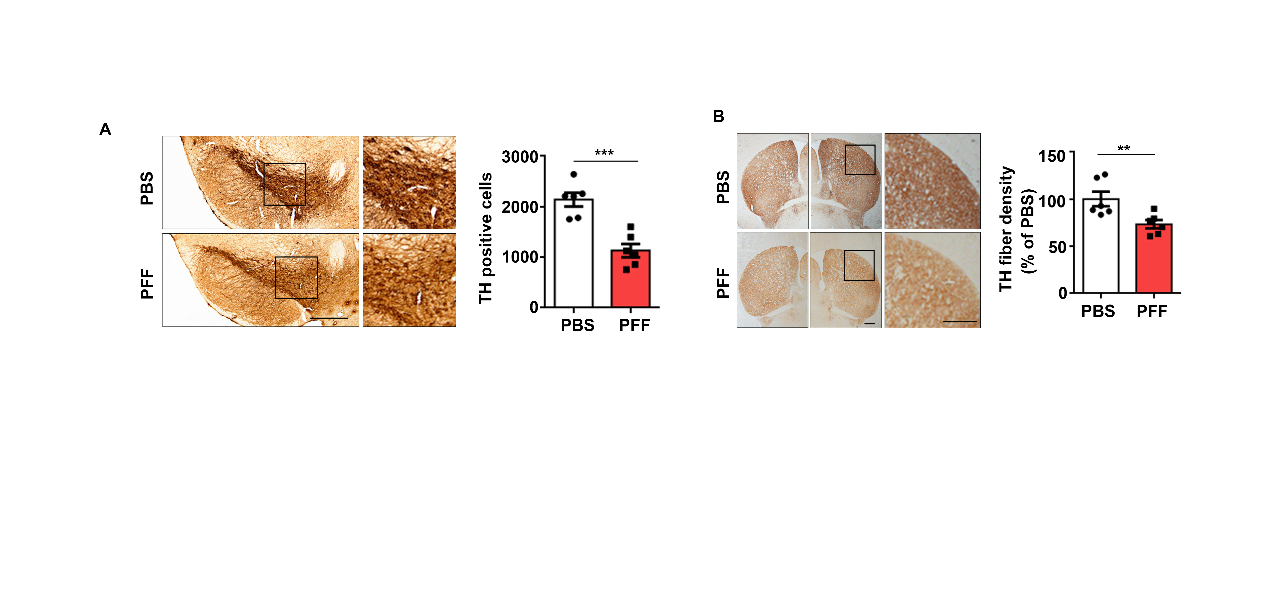


**Figure S10.** Immunohistochemistry staining of tyrosine hydroxylase (TH) in mice injected with α-Syn PFF. (A) Photomicrographs and quantifications of TH-positive cells numbers in SN of mice injected with α-Syn PFF at 3 mpi (n = 6 mice per group). Scale bar, 200 µm. (B) Micrographs and quantifications of TH fiber density in STR of mice injected with α-Syn PFF at 3 mpi (n = 6 mice per group). Scale bar, 200 μm. Data were expressed as mean ± SEM. Two-tailed Student’s t-tests were used for statistical analysis. **P < 0.01, ***P < 0.001.
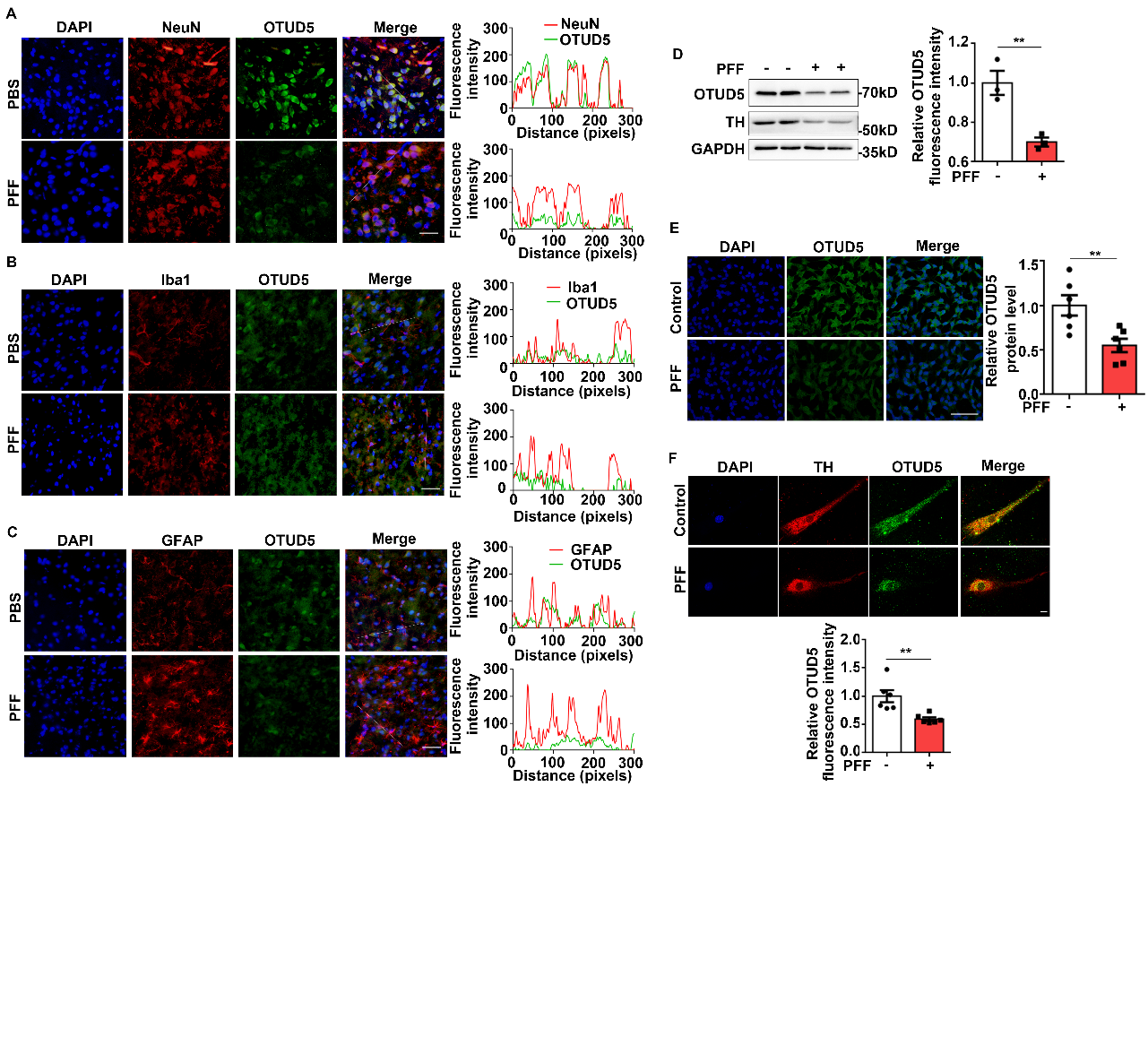


**Figure S11.** OTUD5 was downregulated in PD model induced by α-Syn PFF. (A-C) Photomicrographs of double immunostaining for OTUD5 (green) and NeuN (neuron marker, red), Iba1 (microglia marker, red) or GFAP (astrocyte marker, red) in mouse brain after α-Syn PFF treatment (n=6 mice per group). Intensity profiles of indicated proteins along the plotted lines were analyzed by ImageJ line scan analysis. Scale bar, 20 μm. (D) Immunoblot analysis of OTUD5 expression in SH-SY5Y cells treated with α-Syn PFF. Quantification of α-Syn levels in SH-SY5Y cells were normalized to GAPDH (n = 6 biologically independent). (E) Representative images of immunostaining for OTUD5 (green) in SH-SY5Y cells treated with α-Syn PFF. DAPI (blue) was used for nuclei staining (n = 3 biologically independent). Scale bar, 100 μm. (F) Representative images of OTUD5 (green) and TH (red) in primary cultured midbrain neurons incubated with α-Syn PFF. DAPI (blue) was used for nuclei staining. Quantification of OTUD5 signals normalized with control group (n = 6 biologically independent). Scale bar, 10 μm. Data were expressed as the mean ± SEM. Two-tailed Student’s t-tests were used for statistical analysis in (D-F). **P < 0.01.

**
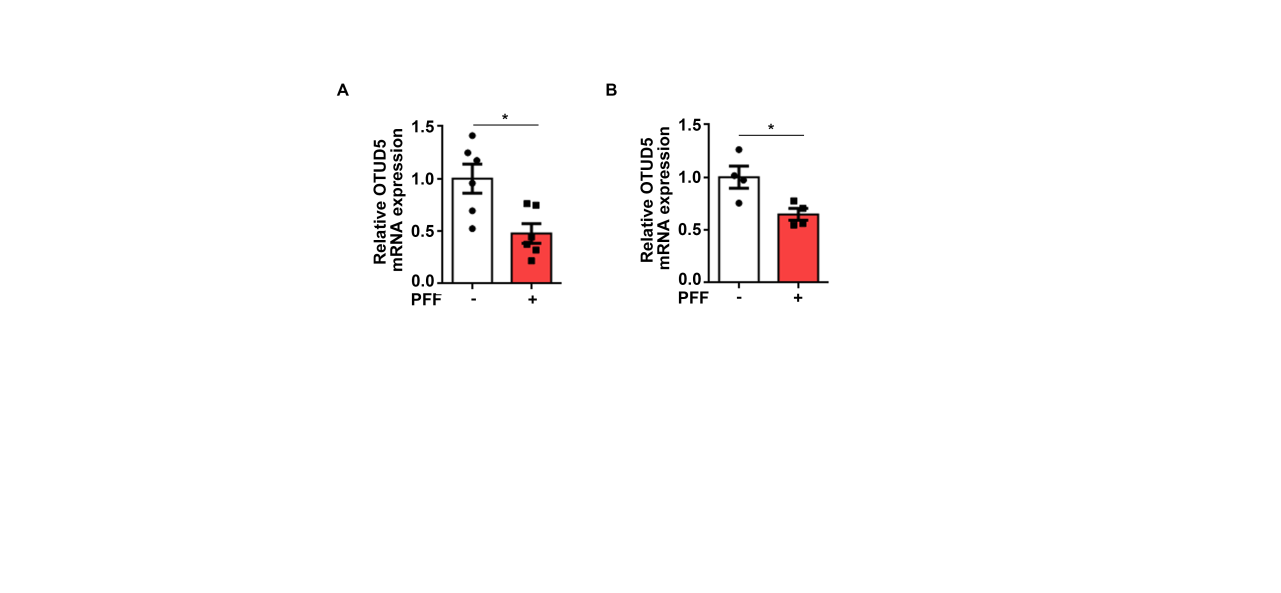
**

**Figure S12.** The mRNA levels of OTUD5 were reduced in PD models induced by α-Syn PFF. (A) Relative mRNA levels of OTUD5 in the SN of brain from α-Syn PFF-injected mice. Quantification of OTUD5 mRNA levels were normalized to β-actin (n = 6 mice per group). (B) Relative mRNA levels of OTUD5 in SH-SY5Y cells stimulated with α-Syn PFF. Quantification of OTUD5 mRNA levels were normalized to β-actin (n = 4 biologically independent). Data were expressed as mean ± SEM. Two-tailed Student’s t-tests were used for statistical analysis. *P < 0.05.


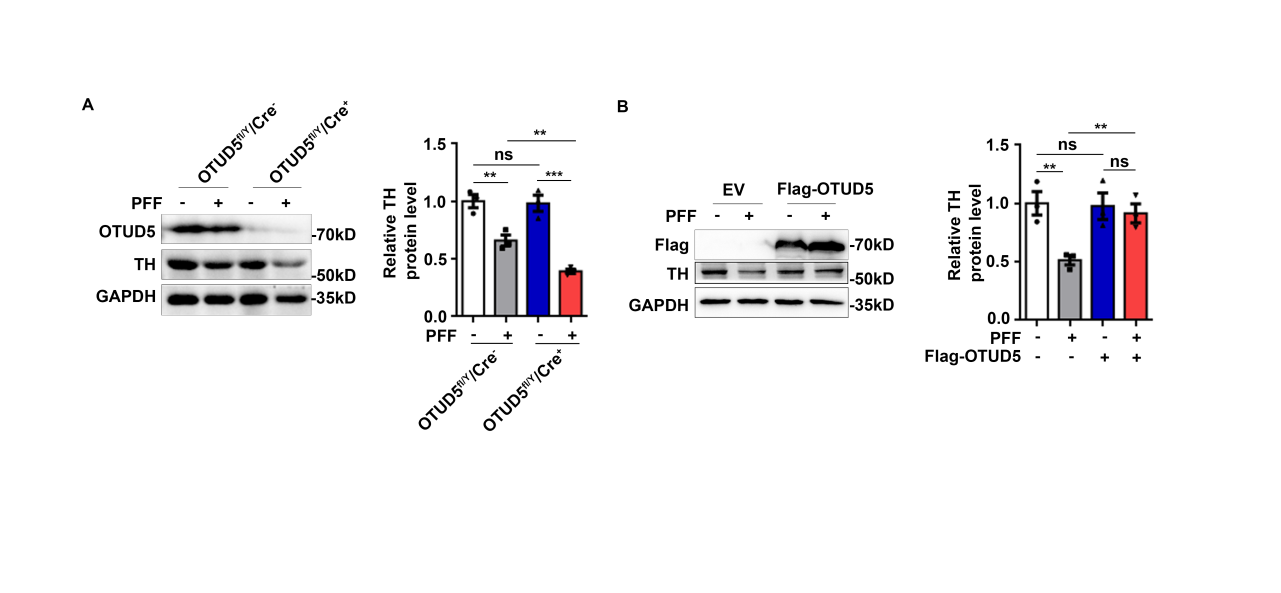
­­

**Figure S13.** OTUD5 protected against α-Syn PFF-induced neurondegenration. (A) Immunoblot analysis and quantification of lysates from primary cultured midbrain neurons of OTUD5^fl/Y^/Cre^-^ and OTUD5^fl/Y^/Cre^+^ mice treated with PBS or α-Syn PFF. Quantification of TH levels were normalized to GAPDH (n = 3 biologically independent). The interaction between OTUD5 CKO and α-Syn PFF was statistically significant (P = 0.043). (B) Immunoblot analysis and quantification of lysates from SH-SY5Y cells transfected with Flag-OTUD5 expression plasmid and then incubated with α-Syn PFF. Quantification of TH levels were normalized to GAPDH (n = 3 biologically independent). The interaction of Flag-OTUD5 and α-Syn PFF was statistically significant (P = 0.042). Data were expressed as the mean ± SEM. Two-way ANOVAs for 2*2 factorial analysis and Tukey’s post hoc tests were used for statistical analysis. *P < 0.05, **P < 0.01.


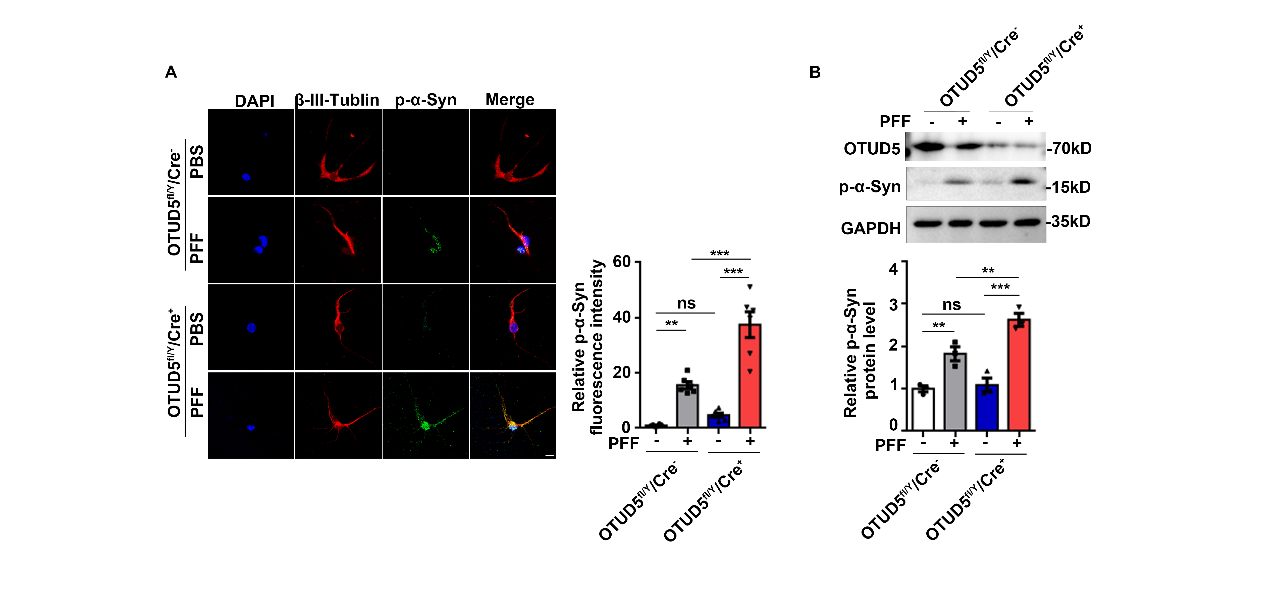


**Figure S14.** OTUD5 decreased p-α-Syn level induced by α-Syn PFF. (A) Representative microscopic images of p-α-Syn (Ser129) (green) in primary cultured midbrain neurons of OTUD5^fl/Y^/Cre^-^ and OTUD5^fl/Y^/Cre^+^mice treated with PBS or α-Syn PFF. DAPI (blue) is used for nuclei staining. Quantification of p-α-Syn (Ser129) immunofluorescence intensity was normalized to PBS group (n = 6 biologically independent). The interaction between OTUD5 CKO and α-Syn PFF was statistically significant (P = 0.001). Scale bar, 10 μm. (B) Immunoblot analysis and quantification of lysates from primary cultured midbrain neurons of OTUD5^fl/Y^/Cre^-^ and OTUD5^fl/Y^/Cre^+^ mice treated with PBS or α-Syn PFF (n = 3 biologically independent). The interaction of OTUD5 CKO and α-Syn PFF was statistically significant (P = 0.038). Data were expressed as the mean ± SEM. Two-way ANOVA for 2*2 factorial analysis and Tukey’s post hoc tests were used for statistical analysis. **P < 0.01, ***P < 0.001.

**Table S1. List of oligonucleotide primers**

| **The sequences of siRNAs used in the study** | **Forward (5’-3’)** | **Reverse (5’-3’)** |
| --- | --- | --- |
| OTUD5 siRNA | AGCCUUCAAUGUUGAAUUUUU | AAAAAUUCAACAUUGAAGGCU |
| NEDD4 siRNA | CAAGCACAACGUGCAUUUATT | UAAAUGCACGUUGUGCUUGCA |
| **The sequences of sgRNAs used in the study** | **Forward (5’-3’)** | **Reverse (5’-3’)** |
| OTUD5 sgRNA#1 | CACCGCGACCGTGACTCCGGCGTCG | AAACCGACGCCGGAGTCACGGTCGC |
| OTUD5 sgRNA#2 | CACCGGGCGATCGCGACCGTGACTC | AAACGAGTCACGGTCGCGATCGCCC |
| OTUD5 sgRNA#3 | CACCGCGCCGGGCGCTCTTCATCGC | AAACGCGATGAAGAGCGCCCGGCGC |
| ATG5 sgRNA | CACCGTGATATAGCGTGAAACAAGT | AAACACTTGTTTCACGCTATATCAC |
| **Primer pairs used for RT-PCR** | **Forward (5’-3’)** | **Reverse (5’-3’)** |
| human SNCA | GCAGAAGCAGCAGGAAAGAC | ACCACTGCTCCTCCAACATT |
| human OTUD5 | GGTTGTGCGAAAGCATTGCAT | ACCTCCACAGGACGGTTGT |
| mouse OTUD5 | CTGCCTTTGGTCTGAATGATTG | GTCTAGGTATTCCTGTTGGGAC |
| human β-actin | CATGTACGTTGCTATCCAGGC | CATGTACGTTGCTATCCAGGC |
| mouse β-actin | GGCTGTATTCCCCTCCATCG | CCAGTTGGTAACAATGCCATGT |
| **Primer pairs used for PCR** | **Forward (5’-3’)** | **Reverse (5’-3’)** |
| OTUD5^flox/flox^ | TTCTACCCATTTCCTGTAGTGG | CTAATTCATGTCCCATCATAACCTA |
| DAT-Cre | TGGCTGTTGGTGTAAAGTGG | CCAAAAGACGGCAATATGGT |
